# BG4 antibody can recognize telomeric G-quadruplexes harboring destabilizing base modifications and lesions

**DOI:** 10.1093/nar/gkad1209

**Published:** 2023-12-28

**Authors:** Samuel A Johnson, Tapas Paul, Samantha L Sanford, Brittani L Schnable, Ariana C Detwiler, Sanjana A Thosar, Bennett Van Houten, Sua Myong, Patricia L Opresko

**Affiliations:** Department of Environmental and Occupational Health, University of Pittsburgh School of Public Health, Pittsburgh, PA 15261, USA; UPMC Hillman Cancer Center, Pittsburgh, PA 15213, USA; Molecular Biophysics and Structural Biology Graduate Program, University of Pittsburgh, PA 15260, USA; Department of Biophysics, Johns Hopkins University, Baltimore, MD 21218, USA; Program in Cellular and Molecular Medicine, Boston Children's Hospital, Boston, MA 02115, USA; Department of Environmental and Occupational Health, University of Pittsburgh School of Public Health, Pittsburgh, PA 15261, USA; UPMC Hillman Cancer Center, Pittsburgh, PA 15213, USA; UPMC Hillman Cancer Center, Pittsburgh, PA 15213, USA; Molecular Biophysics and Structural Biology Graduate Program, University of Pittsburgh, PA 15260, USA; Department of Environmental and Occupational Health, University of Pittsburgh School of Public Health, Pittsburgh, PA 15261, USA; UPMC Hillman Cancer Center, Pittsburgh, PA 15213, USA; Department of Environmental and Occupational Health, University of Pittsburgh School of Public Health, Pittsburgh, PA 15261, USA; UPMC Hillman Cancer Center, Pittsburgh, PA 15213, USA; UPMC Hillman Cancer Center, Pittsburgh, PA 15213, USA; Molecular Biophysics and Structural Biology Graduate Program, University of Pittsburgh, PA 15260, USA; Department of Pharmacology and Chemical Biology, University of Pittsburgh School of Medicine, PA 15213, USA; Department of Biophysics, Johns Hopkins University, Baltimore, MD 21218, USA; Program in Cellular and Molecular Medicine, Boston Children's Hospital, Boston, MA 02115, USA; Department of Environmental and Occupational Health, University of Pittsburgh School of Public Health, Pittsburgh, PA 15261, USA; UPMC Hillman Cancer Center, Pittsburgh, PA 15213, USA; Molecular Biophysics and Structural Biology Graduate Program, University of Pittsburgh, PA 15260, USA; Department of Pharmacology and Chemical Biology, University of Pittsburgh School of Medicine, PA 15213, USA

## Abstract

BG4 is a single-chain variable fragment antibody shown to bind various G-quadruplex (GQ) topologies with high affinity and specificity, and to detect GQ in cells, including GQ structures formed within telomeric TTAGGG repeats. Here, we used ELISA and single-molecule pull-down (SiMPull) detection to test how various lengths and GQ destabilizing base modifications in telomeric DNA constructs alter BG4 binding. We observed high-affinity BG4 binding to telomeric GQ independent of telomere length, although three telomeric repeat constructs that cannot form stable intramolecular GQ showed reduced affinity. A single guanine substitution with 8-aza-7-deaza-G, T, A, or C reduced affinity to varying degrees depending on the location and base type, whereas two G substitutions in the telomeric construct dramatically reduced or abolished binding. Substitution with damaged bases 8-oxoguanine and O6-methylguanine failed to prevent BG4 binding although affinity was reduced depending on lesion location. SiMPull combined with FRET revealed that BG4 binding promotes folding of telomeric GQ harboring a G to T substitution or 8-oxoguanine. Atomic force microscopy revealed that BG4 binds telomeric GQ with a 1:1 stoichiometry. Collectively, our data suggest that BG4 can recognize partially folded telomeric GQ structures and promote telomeric GQ stability.

## Graphical Abstract

**Graphical Abstract:**
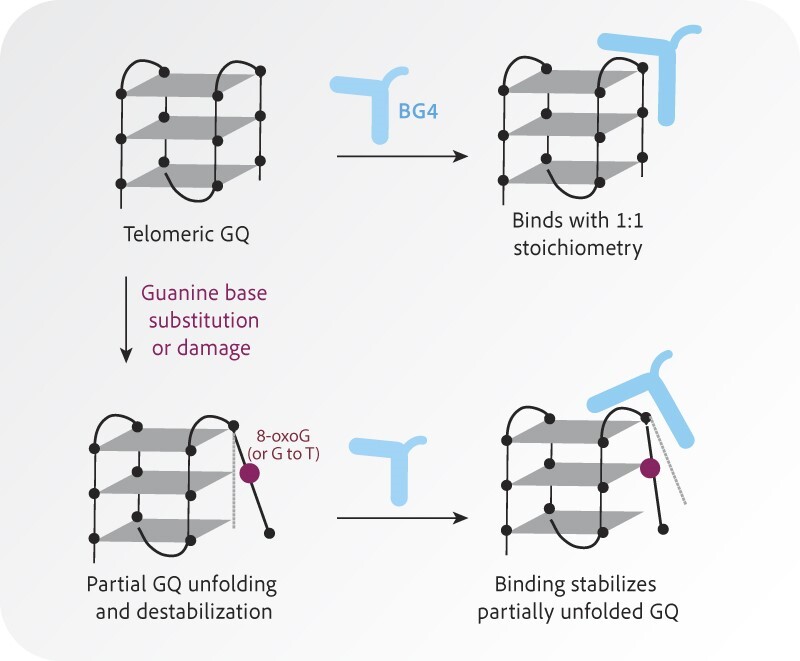

## Introduction

Telomeric nucleoprotein caps at chromosomal ends in humans consist of repetitive TTAGGG duplex DNA followed by a 50–500 G-rich nucleotide 3′ single-stranded overhang, and are coated by a complex of proteins, collectively termed shelterin (1). Telomeres prevent the chromosome ends from being falsely recognized as DNA double-strand breaks by remodeling into a t-loop structure in which the overhang invades duplex telomeric DNA forming a displacement loop (D-loop) (2–4). Telomere attrition during cell division eventually leads to irreversible growth arrest, termed senescence, or genomic instability through chromosome end fusions (5). The enzyme telomerase compensates for telomere loss by binding the 3′ overhang and uses an integrated RNA template to add GGTTAG repeats to the overhang (6,7). The majority of cancers upregulate telomerase to achieve unlimited proliferation, while about 15% use the alternative lengthening of telomeres (ALT) pathway which hijacks homology-directed repair mechanisms to restore telomeres (8–10). Therefore, telomere maintenance is critical for sustained cellular proliferation and genome stability.

The G-rich nature of telomeric repeats allows them to fold spontaneously into G-quadruplex (GQ) structures which are implicated in multiple aspects of telomere biology. GQs are non-canonical secondary structures that can form in single-stranded (ss) DNA or RNA containing repetitive guanine runs, in which four guanines form planar G-quartets through Hoogsteen bonding. Monovalent cations, Na^+^ or K^+^, residing within the central radial axis of the GQ stabilize the structure (11). Telomeric GQ consists of three stacked quartets and can arise within a single ssDNA molecule, or multiple molecules, and form various hybrid-type conformations of mixed one parallel and three anti-parallel strands in K^+^ solutions (12,13). GQs have been proposed as a rudimentary alternative cap to the t-loop structure, particularly during S-phase when the t-loop is resolved (14). However, GQ formation on the G-rich lagging strand can impair telomere replication, leading to telomere fragility or losses, which are prevented by DNA helicases including BLM and RTEL1 that unwind GQs (15,16). GQ folding in the telomeric ssDNA 3′overhang, and ligands that stabilize GQs, can inhibit telomerase loading and telomere extension (17), but GQ folding in the telomerase product DNA promotes telomerase translocation and its extension rate (18). GQs and GQ ligands can facilitate ALT-mediated telomere extension by causing replication stress which provokes homology-directed repair (19,20), and GQs are enriched at ALT telomeres (21). Yet, ALT cells are sensitive to killing by GQ ligands (19,22), suggesting stable GQ structures may compromise productive ALT. Therefore, factors that positively or negatively regulate GQ folding at telomeres can profoundly influence maintenance and stability.

Structural and biophysical studies demonstrate that damaged DNA bases and base substitutions within telomeric sequences alter GQ properties by reducing Hoogsteen bonding, thereby increasing structural dynamics and accessibility without inducing complete unfolding (23). Telomeric sequences are hypersensitive to oxidative stress and formation of the common oxidative lesion 8-oxoguanine (8-oxoG) due to the G-rich sequence (24). An 8-oxoG decreases the GQ melting temperature from 65°C to below 50°C, however, NMR data reveal that minor structural rearrangements or major conformation shifts can accommodate an 8-oxoG in the GQ depending on the lesion location (25). Using single molecule FRET (smFRET) we showed that introducing a single 8-oxoG increases GQ structural dynamics, as well as the loading and activity of telomerase and ALT-related protein RAD51 (26–28). Alkylation lesion O6-methylation guanine imparts greater GQ instability than 8-oxoG (26). GQ folding also impacts base excision repair since 8-oxoguanine glycosylase (OGG1) repair enzyme cannot excise 8-oxoG in a telomeric GQ, and APE1 activity is absent or reduced (29). Base substitutions at the G residues also induced GQ instability depending on the base type and location (26). Collectively, these studies reveal that single-base modifications drive structural alterations that influence repair and extension activity in telomeric DNA.

Although base damage and substitution alter telomeric GQ folding properties *in vitro*, whether they alter GQ abundances at telomeres in cells remains unknown. GQs in cells can be detected by immunofluorescence with the GQ specific antibody BG4, which binds a wide range of DNA and RNA GQ conformations and detects GQ enrichment in S-phase cells, after GQ ligand addition, and depletion of helicases (30–32). Conversely, whether BG4 can detect a reduction in GQs at telomeres due to disruptive base modification depends on the extent to which base changes alter BG4 affinity. To address this, we tested an array of telomeric substrates containing site-specific base substitutions and lesions for BG4 binding by complementary enzyme-linked immunosorbent assay (ELISA) and single-molecule pull down (SiMPull). We found BG4 retained high affinity (4–5 nM) for telomeric GQ containing 4 or more TTAGGG repeats, but exhibited a ∼2–3-fold reduction in affinity for constructs with three TTAGGG repeats which cannot fold into unimolecular GQ. Disrupting a Hoogsteen bond with guanine base substitutions or lesions in a single repeat reduced BG4 affinity, but disrupting multiple bonds with substitutions in two repeats nearly abolished binding, suggesting BG4 can bind to partially folded, but not unfolded, GQ structures. SiMPull combined with smFRET demonstrated that BG4 binding can promote folding of telomeric GQ harboring a single G replaced with T or 8-oxoG at a single repeat. We further show 1:1 stoichiometry of BG4 binding to unimolecular telomeric GQ. Our studies reveal that BG4 can recognize partially folded telomeric GQ structures, and promote telomeric GQ stability.

## Materials and methods

### DNA oligonucleotide and labeling

The HPLC purified unlabeled and Cy3 or Cy5 labeled oligonucleotides used in this study (Supplementary Tables 1–3) were synthesized by Integrated DNA Technologies except the oligonucleotides containing 8-oxoguanine (8-oxoG) and O6-methylguanine (O6meG) for the single molecule studies (Supplementary Table 2) were synthesized by Midland Certified Reagent Company Inc (Midland, TX). The oligonucleotides with an 8-oxoG and O6meG, also contained a primary amine modification at the designated labeling site and were labeled with NHS-ester conjugated fluorescent dye as described previously (27). In brief, oligonucleotides (40 μM) were reacted with 3.3 mM Cy3-NHS ester (GE Healthcare) in 100 mM sodium bicarbonate buffer overnight at room temperature. Excess dye was removed by ethanol precipitation and the labeling efficiency at ∼80–90% for all oligonucleotides containing a damaged base was calculated using a NanoDrop spectrophotometer. To prepare the partial duplex constructs, G-rich ‘top-strand’ oligonucleotides were mixed in 20 mM Tris pH 7.5, 100 mM KCl, with a 1.5-fold excess of the 18-mer ‘stem complement’ oligonucleotide. Oligonucleotides were then annealed and folded into G-quadruplex by incubating at 90° to 95°C for 2–10 min and then slow cooling to room temperature.

### BG4 expression and purification

C-terminal tagged 6x-histidine and 3x-FLAG BG4 protein was expressed and purified as described previously (31) with some modification. BG4/pSANG10-3F plasmid was a gift from Brad Johnson (University of Pennsylvania) and was transformed into BL21 DE3 *Escherichia coli* (New England Biolabs). The 5-ml starter bacteria culture (10 g/l N-Z amine AS, 5 g/l yeast extract, 2% glucose, 50 μg/ml kanamycin) was incubated for 16 h overnight at 37°C. Then, the culture was added to 250 ml auto-induction media (10 g/l N-Z amine AS, 5 g/l yeast extract, 2 mM MgSO_4_, 0.5% glycerol, 0.2% lactose, 0.05% glucose, 25 mM KH_2_PO_4_, 50 mM NH_4_Cl, 5 mM Na_2_SO_4_), and incubated 6 h at 37°C, then 16 h overnight at 25°C. Cells were harvested by centrifugation and frozen at −80°C. Cell pellets were thawed on ice and lysed by resuspension in 20 ml TES lysis buffer (50 mM Tris pH 8.0, 1 mM EDTA, 20% sucrose) for 10 minutes on ice. 30 ml of 1:5 TES lysis buffer (PBS with 10 mM imidazole, 10% glycerol and 1 dissolved cOmplete™ protease inhibitor tablet (Roche)) was added and incubated for 15 min on ice. Lysate was centrifuged 10 min at 5250 × g, and supernatant was collected and filtered through 0.2-μm PES membrane filter (Corning).

Column chromatography was used to purify BG4 from lysate on an ÄKTA Pure FPLC (Cytiva). All buffers included a cocktail of protease inhibitors consisting of 2 μg/ml leupeptin, 2 μg/ml aprotinin, 2 μg/ml pepstatin, 2 μg/ml chymostatin and 1 mM AEBSF. Protein was captured on a 5-ml HisTrap FF affinity column (Cytiva) by adding filtered supernatant at a rate of 1 ml/minute, then washed with 5 column volumes of low imidazole buffer (10 mM imidazole, 5 mM β-mercaptoethanol, 10% glycerol in PBS). Protein was eluted with high imidazole buffer (250 mM imidazole, 5 mM β-mercaptoethanol, 10% glycerol in PBS) in 1 ml fractions. Fractions corresponding to and flanking the UV absorbance peak were resolved by gel electrophoresis and visualized with Coomassie InstantBlue™ stain (Sigma-Aldrich). Fractions with a strong band corresponding to BG4 32.7-kDa were selected, pooled, and concentrated to a total volume of 500 μL using Amicon Ultra-4 10-kDa centrifugal filters (Millipore). 4 ml Superdex buffer (25 mM Tris pH 7.5, 150 mM sodium chloride, 5 mM DTT, 5% glycerol) was added and pooled fractions were concentrated again to approximately 900 μl using Amicon Ultra-4 10-kDa centrifugal filters (Millipore).

Protein was filtered through a 0.2 μm cellulose filter (Cytiva) and loaded onto a Superdex 75 Increase 10/300 GL size exclusion column (GE Healthcare) by loop injection. Protein was eluted with Superdex buffer in 300 μl fractions. Fractions corresponding to and flanking the UV absorbance peak were resolved by gel electrophoresis and visualized with Coomassie InstantBlue™ stain (Sigma-Aldrich). Fractions with a strong band corresponding to BG4 32.7-kDa were selected, pooled, and concentrated to a total volume of 100 μl using Amicon Ultra-4 10-kDa centrifugal filters (Millipore) (Supplementary Figure 1A). The buffer of pooled fractions was exchanged three times for phosphate-buffered saline (PBS) with 5% glycerol using Amicon Ultra-4 10-kDa centrifugal filters (Millipore). Protein was concentrated to 100 μl, divided into 10-μl aliquots, snap-frozen in dry ice and ethanol, and stored at −80°C. Protein concentration was quantified with Pierce^TM^ Coomassie Plus Bradford assay (Thermo Scientific™) according to the manufacturer's instructions.

To determine the estimated molecular weight and stoichiometry of purified BG4 in solution, we prepared a set of known protein standards (Bio-Rad) along with BSA and aprotinin. The protein standard mix (1.625 mg/ml) in 1ml elution buffer (25 mM Tris, pH 7.5, 150 mM NaCl, 5% glycerol) was loaded on a Superdex 75 Increase 10/300 GL column (Cytiva). The elution volume of each protein was recorded. To construct the calibration curve, the gel phase distribution coefficient (*K*_av_) was calculated for each protein standard by *K*_av_ = (*V*_e_ – *V*_o_)/(*V*_c_ – *V*_o_), in which *V*_o_ is the void volume of the column, *V*_c_ is the accessible volume of the column, and *V*_e_ is the analyte retention volume. These values were plotted against the Log_10_ of each molecular weight. The estimated size of BG4 was 34.3 kDa based on the standard curve and its calculated *K*_av_ (Supplementary Figure 1A).

### BG4 immunofluorescence

HeLa LT FAP-TRF1 and U2OS FAP-TRF1 cells (33) were cultured in Dulbecco's modified Eagle medium (DMEM, Gibco) with 10% fetal bovine serum (FBS), 50 units/ml penicillin, and 50 units/ml streptomycin at 37ºC in 5% CO_2_ and 5% O_2_. To generate U2OS FAP-TRF1 OGG1 knock out clones, 293T cells were transfected with pLentiCRISPR V2 plasmids encoding guide RNAs targeting OGG1 exon 4 (gRNA3, sequence GCTACGAGAGTCCTCATATG) and *Streptococcus pyogenes* Cas9 (GeneScript). U2OS FAP-TRF1 cells were infected with lentivirus and selected with 1.5 mg/ml puromycin (Gibco) as described previously (33). After selection, and death of uninfected cells, the infected cells were expanded, and expression of OGG1 was determined by western blotting (anti-OGG1 Abcam ab124741; 1:500) as described (34). Cells were grown on glass coverslips in 35 mm 6-well plates. For GQ-ligand treatment, cells were incubated with 10 μM pyridostatin trifluoroacetate (PDS) (Sigma-Aldrich) for 14 h at 37ºC. For genome-wide 8-oxoG production, cells were treated with 10 or 20 mM KBrO_3_ oxidant for one hour in Opti-MEM (Gibco). Telomere specific 8-oxoG was induced by pre-treating U2OS FAP-TRF1 cells with Opti-MEM for 15 minutes at 37°C, followed by incubation with 100 nM MG2I dye for 15 min. Cells were then exposed to a high intensity 660 nm LED light at 140 mW cm^–2^ for 10 min as previously described (33,35). After 8-oxoG induction, cells were extracted by treating on ice with ice-cold CSK buffer (100 mM NaCl, 3 mM MgCl_2_, 300 mM glucose, 10 mM Pipes pH 6.8, 0.5% Triton X-100, 5 mg/ml RNase A, and protease inhibitors tablet (Roche)). All cells were fixed for 15 min with 2–4% formaldehyde in PBS at room temperature, then washed with PBS and permeabilized for 10 min with 0.2% Triton X-100 in PBS at room temperature. Non-CSK treated samples were incubated for 30 min with 5 mg/ml RNase A (Invitrogen) in PBS at 37ºC. All samples were blocked for 1 h with blocking solution (10% normal goat serum, 1% bovine serum albumin (BSA), and 0.05 or 0.1% Triton X-100 in PBS) at room temperature. Cells were incubated either for 1 h at 37ºC or overnight at 4ºC with BG4 (10 nM) alone or with TRF2 (1:500 dilution, Novus NB 110–57130) as indicated. Cells were washed three times with PBS or PBS-T for 5 min at room temperature and briefly with 1% BSA in PBS. Cells were incubated with rabbit or mouse α-FLAG tag antibody (1:500 dilution, Cell Signaling), for 1 h at 37ºC, and then washed three times with PBS or PBS-T for 5 min at room temperature and briefly with 1% BSA. Then cells were incubated with either goat α-rabbit Alexa 594 antibody alone or together with goat α-mouse Alexa 647 or (1:1000 dilution, Invitrogen) for 1 h at room temperature. Cells were washed three times with PBS or PBS-T for 5 min at room temperature and stained for 10 min with 0.1 mg/ml DAPI in PBS at room temperature. Coverslips were rinsed with PBS and then with water before mounting on slides with Pro-Long Diamond Antifade mountant (Invitrogen). Images of slides were captured with a Nikon Ti inverted fluorescence microscope and processed with Nikon NIS-Elements AR software. 0.2 μm Z-stacks were imaged, deconvolved and maximum intensity projections used for the final images. Exposure times were identical for all images in a given experiment. For BG4 colocalization with telomeres, the object counts feature in NIS AR was used to set a threshold for foci that was kept constant throughout the experiment. The binary function was used to determine the intersections of Cy3 and Cy5 channels in defined regions of interest (ROI) (DAPI-stained nuclei).

### BG4 affinity analysis by ELISA

Oligonucleotides used for ELISA are shown in Supplementary Table 1 and were resuspended at 100 μM in DEPC-filtered water. All washes were performed three times for 5 min each with 1× ELISA wash buffer (Invitrogen™ catalog #WB01). Pierce™ Neutravidin™-coated 96-well clear polystyrene plates (Thermo Scientific catalog #15123) were washed three times. After annealing and folding, oligonucleotide substrates were diluted to 10 nM in 1× ELISA assay buffer (Invitrogen™ catalog #DS98200). Substrates (100 μl each) were added to wells and incubated for 1 h at room temperature with gentle rocking. Wells were aspirated and washed three times. Wells were blocked for one hour with 150 μl blocking buffer (1% BSA, 0.05% Triton X-100, in PBS) with rocking. BG4 dilutions were prepared (0.1 to 40 nM) in 1x ELISA assay buffer. Blocking solution was aspirated and 100 μl BG4 dilutions were added to each well and incubated for one h at room temperature with rocking. Wells were aspirated and washed three times. HRP-conjugated anti-FLAG tag antibody (Abcam ab1238) was diluted 1:50 000 in 1× ELISA assay buffer, and 100 μl was added to each well for 30 min at room temperature with rocking. Wells were aspirated and washed again three times. To visualize binding, 100 μl TMB chromogen (Invitrogen™ catalog #002023) was added to wells and incubated for 4 min at room temperature. The colorimetric reaction was stopped with the addition of 100 μl ELISA stop solution (Invitrogen™ catalog #SS03). Absorbance at 450 nm was measured on a Biotek Synergy 2 plate reader.

Data analysis was performed in GraphPad Prism. Absorbance values for each condition were plotted vs. BG4 concentration and data were fit by non-linear regression to a hyperbolic curve of form $A450 = \frac{{{A}_{max}[ {BG4} ]}}{{{K}_d\ + \ [ {BG4} ]}} + \ NS[ {BG4} ] + B$, where *A*450 is the absorbance at 450 nm, *A*_max_ is the mean maximum binding for the experiment, [BG4] is the concentration of BG4, NS is a variable corresponding to concentration-dependent non-specific binding, and *B* is the background absorbance observed in controls without BG4 added. *A*_max_ was determined for each experiment and defined for each curve in that experiment, and NS was constrained to be equal for all conditions in an experiment. K_d_, the apparent dissociation constant for each reaction, was calculated from these fit curves and reported as a measure of relative binding affinity.

### BG4 affinity analysis by SiMPull

The oligonucleotides used for single-molecule pull-down (SiMPull, non-biotinylated DNA) and single-molecule FRET (smFRET, biotinylated DNA) assay are listed in Supplementary Table 2. The SiMPull and smFRET assay for BG4 binding was performed using custom-built prism-type TIRF microscopy at room temperature (25°C ± 2) as described earlier (36). The details of instrumentation and PEGylated slide preparation were well documented previously (37,38). The SiMPull BG4 binding assay was performed as follows. Initially, the biotinylated mouse anti-FLAG monoclonal M2 antibody (F9291, Sigma) was diluted ∼500 times in T50 buffer (10 mM Tris–HCl, pH 7.5, 50 mM NaCl) and immobilized in the imaging chamber via biotin−NeutrAvidin interaction. The imaging chamber was washed with buffer containing 10 mM Tris–HCl, pH 7.5, 100 mM KCl. The surface was further blocked by biotin (1 mM), BSA (0.4 mg/ml) and yeast t-RNA (0.2 mg/ml) to avoid nonspecific binding of DNA samples as described previously (38,39). Diluted (1–5 nM) FLAG-tagged BG4 antibody was added to the immobilized antibody-coated surface and incubated for 10 min at room temperature. Subsequently, washed out the sample chamber and 10 nM non-biotinylated Cy3-labeled (or both Cy3 and Cy5 labeled) partial duplex DNA was applied to the sample chamber and incubated for 15 min. Unbound DNA was washed out and the imaging buffer containing 10 mM trolox, 0.5% glucose, 1 mg/ml glucose oxidase, and 4 mg/ml catalase with 10 mM Tris–HCl, pH 7.5, 100 mM KCl was added to the sample chamber for imaging. Solid-state 532 nm diode laser (Compass 315M; Coherent, Santa Clara, CA) was used to excite the Cy3 molecules and thirty or more short (∼2 s) movies were recorded from different imaging surfaces. The recorded data were analyzed with a MATLAB script and the binding ratio was calculated for different DNA constructs. The experiment was repeated at least three times on different days, and the average binding ratio and standard error were calculated. In each set of SiMPull BG4 binding assays, we conducted two controls lacking either the anti-FLAG antibody or BG4 protein, along with one standard sample of TELO4, which can form a single folded GQ. The binding affinity was calculated based on the Cy3 foci count of the individual bound DNA molecules. For the custom labeled DNA, BG4 bound molecules were further calculated based on the labeling efficiency. Hence, the BG4 binding contribution of unlabeled damage GQ has been considered. The relative binding affinity of each individual construct is reflected by comparison with the Cy3 foci count of the standard TELO4 construct included in each experiment.

### Single-molecule FRET and data analysis

Each partial duplex DNA construct (10 μM) was prepared by mixing the appropriate pairs of ssDNA and annealed them in a buffer containing 10 mM Tris–HCl, pH 7.5 and 100 mM KCl. The DNA mixtures were incubated at 95°C for 2 min, then gradually cooling at the rate of 2°C/min until 40°C is reached, followed by further 5°C/min cooling until 4°C (36,37). Freshly annealed stocks of partial duplex DNA labeled with biotin, Cy3, and Cy5 were diluted to 15–20 pM in buffer solution (10 mM Tris–HCl, pH 7.5 and 100 mM KCl). The diluted partial duplex DNA was immobilized on the PEG-passivated surface via biotin–NeutrAvidin (50 μg/ml) linkage, and unbound DNA molecules are washed out. All smFRET measurements were carried out in presence (150 nM) and absence of BG4 in above mentioned imaging buffer condition. The evanescent field was generated through PTIR using a solid state of either 532 or 634 nm diode laser (Compass 315M, Coherent) to excite the fluorophores (Cy3 or Cy5) at the sample chamber. The fluorescence from Cy3 (donor) and Cy5 (acceptor) were simultaneously collected using a water immersion objective and finally projected onto the EMCCD camera (Andor) after passing through the dichroic mirror (cut off = 630 nm). Data were recorded with 100 ms frame integration time, then processed by IDL script (http://www.exelisvis.co.uk/ProductsServices/IDL.aspx) and finally analyzed by MATLAB scripts (https://www.mathworks.com/). The data were processed and analyzed following standard methods (27,40).

Single-molecule FRET histograms were generated from more than 4000 molecules (21 frames of 20 short movies) collected from different imaging surfaces. The donor-leakage was corrected based on the FRET values of donor-only molecules. The smFRET histograms were normalized and fitted to multi-peak Gaussian distribution with unconstrained peak position in Origin 2018 (https://www.originlab.com/). Before and after BG4 addition, long movies (1200 frames i.e. 120 s) were recorded to look through the molecular behavior using MATLAB script. The dwell time of the high FRET state in the presence and absence of BG4 was calculated by measuring the time the molecule spends in a high-FRET state. The means and standard errors were plotted.

### BG4 binding observed by atomic force microscopy (AFM)

Sequences of oligonucleotides used for AFM are listed in Supplementary Table 3. Deposition buffer (25 mM HEPES pH 7.5, 50 mM KCl, 10 mM MgCl_2_) and binding buffer (20 mM Tris pH 7.5, 100 mM KCl) were filtered through a 0.02 μm Whatman™ Anotop filter (Sigma-Aldrich). Buffers were also incubated 10 min at 65ºC to dissolve crystals and then cooled to room temperature prior to sample preparation. BG4-substrate binding reactions were incubated for 30 min at 37ºC in binding buffer. Substrates and binding reactions were diluted 1:25 in deposition buffer, deposited on freshly cleaved mica for 30 s, rinsed with filtered, autoclaved water, dried under a stream of nitrogen gas, and incubated for at least 30 min at room temperature prior to imaging. AFM images were collected on a Multimode V Microscope with E scanner (Bruker) using a 2-nm radius SCANASYST-AIR silicon nitride tip (Bruker), and the ScanAsyst PeakForce Tapping mode with peak force setpoint at 0.01988 Volts. 1 μm × 1 μm square images were collected with a resolution of 512 × 512 pixels and a scan rate of 0.977 Hz.vAFM images were analyzed in Image SXM. Particle dimensions were determined and particle volume was calculated from the footprint area and height above background. Histograms were plotted using Graphpad Prism and fitted with Gaussian distributions by nonlinear regression to determine mean particle volume. To calculate BG4’s molecular weight by AFM, a calibration curve was constructed with AFM volumes of OGG1, UvrA, and UvrB determined previously (41), with the equation $V = 1.019 \times MW + 5$, where *V* is the AFM volume in nm^3^ and MW is the molecular weight in kilodaltons.

## Results

### Validation of BG4 specificity and affinity for G-quadruplex substrates

We prepared 3xFLAG-tagged BG4 antibody as described (31), but added a size-exclusion chromatography step to enhance BG4 purity and remove any residual nucleic acids. We reasoned that contaminating nucleic acid might interfere with substrate binding, single-molecule analysis and AFM imaging. SDS-PAGE and Coomassie staining shows the high purity of our BG4 preparation (Supplementary Figure 1A). To confirm GQ detection, we performed immunofluorescence (IF) staining after treating HeLa LT cells with 10 μM of the GQ-stabilizing ligand pyridostatin (PDS) overnight (Supplementary Figure 1B, C>). Consistent with previous reports (31), PDS treatment significantly increased the number of nuclear BG4 foci, indicating that our BG4 preparation was active and sensitive to increased formation of stabilized GQ structures in cells.

Next, we conducted ELISA similar to those performed previously (31) to ensure our BG4 preparation has high affinity and specificity for GQ DNA constructs. Biotinylated annealed and pre-folded DNA constructs (Supplementary Table 1) in 100 mM KCl buffer were bound to Neutravidin-coated plates, and incubated with increasing 3x-FLAG tagged BG4 concentrations. Bound BG4 was detected with anti-FLAG antibodies and colorimetric analysis (Supplementary Figure 1D). BG4 showed nanomolar affinity for GQ substrates, binding with apparent *K*_d_ values of 5.0 ± 0.9 nM for telomeric GQ (TELO4) and 6.5 ± 1.6 nM for the KIT1 GQ (Supplementary Figures 1E–G), comparable to previous reports of 1.5–2.0 nM *K*_d_ for BG4 binding to these substrates (31). Single- and double-stranded non-GQ negative controls produced shallow BG4 hyperbolic binding curves yielding apparent *K*_d_ values greater than 45 nM, outside the range of BG4 concentrations assayed, confirming poor interaction. BG4 showed higher affinity for the T_25_ poly-thymidine oligonucleotide with an apparent *K*_d_ of 25 ± 12 nM than for the other negative controls, but lower affinity compared to the GQ substrates (Supplementary Figures 1E-G). Collectively, these results confirm our BG4 preparation shows high specificity for GQ substrates.

### BG4 retains high affinity for telomeric GQ regardless of telomere length

**Figure 1.**
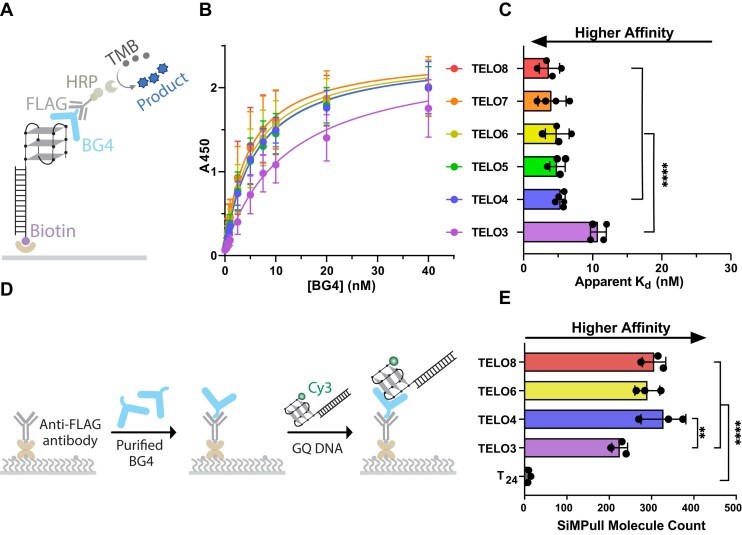
BG4 retains high-affinity telomeric GQ oligonucleotides of different lengths. (**A**) Schematic of ELISA experiment. Construct names indicate the number of repeats; e.g. TELO8 is (TTAGGG)_8_. Complete sequences shown in Supplementary Tables 1 and 2. Telomeric GQ is depicted in an anti-parallel conformation for simplicity. (**B**) ELISA binding curves for GQ constructs that vary in TTAGGG repeat number. Binding is represented by absorbance at 450nm vs. BG4 concentration (nM). (**C**) Apparent dissociation constants (*K*_d_) are calculated from the nonlinear regression curves shown in (**B**). Data are mean ± SD from 4–5 independent experiments; one-way ANOVA. (**D**) Schematic SiMPull assay for binding of DNA constructs to BG4 attached to slides via FLAG antibody. (**E**) Quantification of SiMPull counts of BG4 binding events. Data are mean ± SD from three independent experiments; ordinary one-way ANOVA. Lack of symbol indicates not significant, **P < 0.01, ****P< 0.0001.

To better understand how BG4 binds GQ structures at telomeres in human cells, we asked whether the number of telomeric repeats influences BG4 binding. Human telomeres terminate in a 3′ single-strand (ss) overhang consisting of ∼8–30 TTAGGG repeats (42). We reported previously, using single molecule FRET, that while substrates with four TTAGGG repeats fold into stable GQ, adding more repeats increases the GQ dynamics, conformational states, and accessibility (28). Therefore, we tested a similar series of constructs with identical duplex stems and 3′-ssDNA overhangs consisting of three (TELO3) to eight (TELO8) TTAGGG repeats by ELISA (Figure 1A). We observed a small, although not statistically significant, increase in BG4 affinity as a function of the number of repeats (Figures 1B, C). The apparent K_d_ decreased slightly from 5.4 ± 0.6 nM for TELO4 to 3.7 ± 1.5 nM for TELO8. We and others reported that telomeric oligonucleotides may not fold into the maximum number of GQs, and that TELO8 primarily forms a single GQ (41,43), consistent with the observed similar affinities. BG4 affinity for the TELO3 substrate was significantly reduced with an apparent *K*_d_ of 10.8 ± 1.1 nM, as expected since this construct cannot form a unimolecular GQ. The results show that BG4 affinity for intramolecular telomeric GQ, as measured by ELISA, is not influenced by repeat number.

Bulk biochemistry experiments cannot distinguish between unimolecular and bi-molecular GQ, which BG4 may recognize. Hoogsteen bonding between two telomeric oligonucleotides could form a bi-molecular GQ. Furthermore, although the GQ constructs are bound to the ELISA plates by a duplex stem, the attachment might influence GQ dynamics or properties. Therefore, we examined BG4 binding to telomeric constructs that differ in length at the single molecule level, by conducting single-molecule pull-down (SiMPull). In this technique, we applied 3xFLAG-BG4 protein to single-molecule slides coated with anti-FLAG antibody. Then, we added annealed prefolded, Cy3-labeled DNA constructs (10 nM) lacking biotin and performed TIRF microscopy to capture signals from individual DNA molecules bound to BG4 (Figure 1D). We confirmed that Cy3 fluorescence is not detected on slides lacking anti-FLAG antibody or BG4 (Supplementary Figure 2A-B). The relative binding affinity of each construct to BG4 was calculated from the Cy3 foci count within a region of interest, indicative of individual bound DNA molecules. A single Cy3 photobleaching step indicates that the foci each primarily represent one bound DNA molecule for TELO3 and TELO4, suggesting a lack of bi-molecular GQ (Supplementary Figure 2C). In side-by-side experiments, BG4 showed similar binding to TELO4, TELO6, and TELO8 constructs, averaging approximately 300 foci counted for each (Figure 1E). Compared to these constructs, BG4 showed significantly reduced binding to individual TELO3 molecules yielding a mean foci count of 225 ± 19. As a negative control, BG4 showed very little binding to the T_24_ polythymidine sequence, with a mean of only 11 ± 4 foci. These results indicate BG4 binds to longer telomeric substrates with high affinity, despite altered GQ properties, and suggest BG4 may bind partially folded GQ-like structures in molecules containing three telomeric repeats.

### BG4 binding is specific to GQ structure, not telomeric sequence

**Figure 2.**
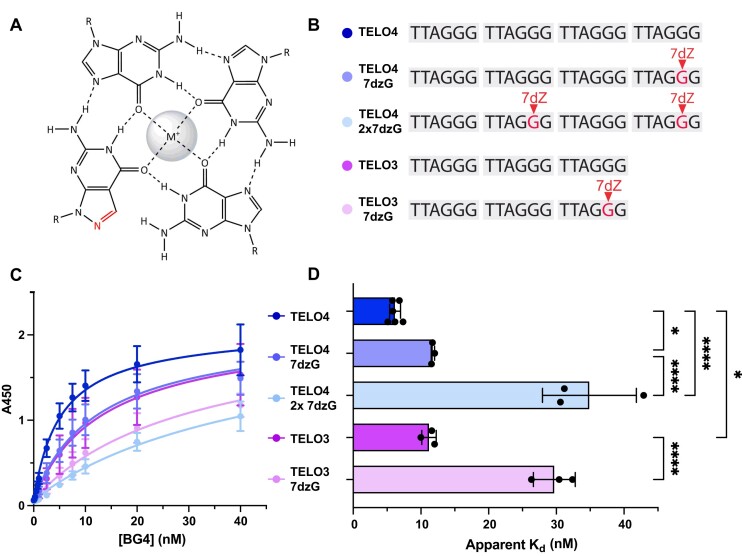
8-aza-7-deazaguanine (7dzG) substitution reduces BG4 affinity for telomeric constructs. (**A**) Chemical structure of 7dzG in a G-quartet demonstrating loss of Hoogsteen bonding with adjacent guanine. (**B**) Schematic of 5′biotinylated DNA constructs containing an 18-base pair stem. Complete sequences shown in Supplementary Table 1. (**C**) ELISA binding curves for 7-dzG-modified telomeric constructs. BG4 binding is represented by absorbance at 450 nm versus the BG4 concentration. (**D**) Apparent dissociation constants (*K*_d_) were calculated from the nonlinear regression curves shown in (**C**). Data are mean ± SD from 3–5 independent experiments; ordinary one-way ANOVA; *P < 0.1, ****P< 0.0001.

To confirm that BG4 binding to telomeric substrates is due to GQ folding, or partial folding, and not to reaction with telomeric sequence, we designed a series of telomeric constructs containing 8-aza-7-deazaguanine (7dzG) substitutions. 7dzG is a guanine analog that retains normal Watson-Crick-Franklin base pairing with cytosine, but eliminates Hoogsteen bonding required for stable GQ formation (Figures 2A) (44–46). A single 7dzG substitution for the 2nd G of the last repeat (TELO4 7dzG) significantly reduced BG4 binding, compared to the unmodified TELO4 substrate (Figures 2B-D). Interestingly, the apparent *K*_d_ for TELO4 with a single 7dzG (11.8 ± 0.2 nM) was similar to that for TELO3 (Figures 1C and 2D). To further disrupt G-quartets and GQ formation we tested a TELO4 construct containing two 7dzG substitutions for the 2nd G in the second and fourth telomeric repeats, as well as a TELO3 substrate with a single 7dzG substitution. BG4 showed comparably poor binding to each substrate, with an apparent *K*_d_ of 41.3 ± 2.2 nM for the TELO4 2×7dzG substrate and 31.8 ± 5.0 nM for the TELO3 7dzG substrate (Figures 2B–D). These results indicate that disruption of Hoogsteen bonds and stable GQ formation impairs BG4 recognition of the telomeric substrates in a manner dependent on the number of disrupted Hoogsteen bonds.

### Base substitution type and position determine the impact on BG4 affinity

**Figure 3.**
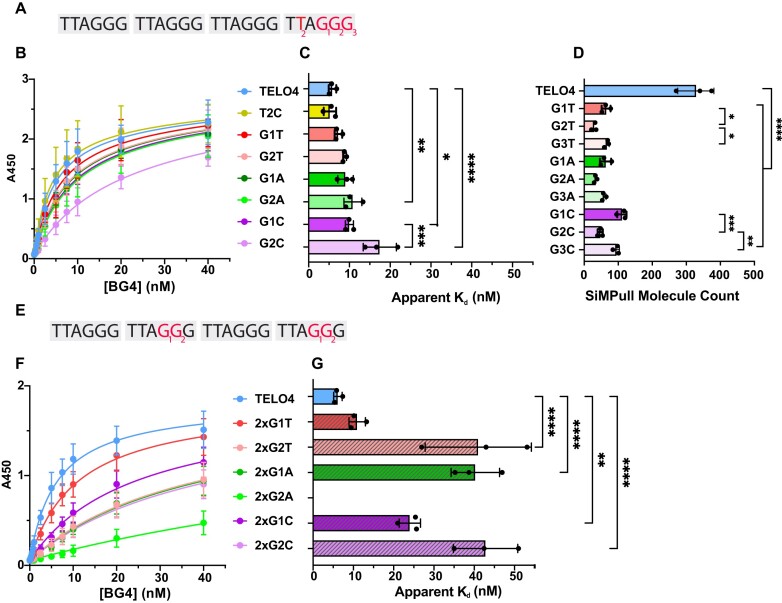
Base substitution type and location determine the impact on BG4 affinity. Constructs are based on TELO4 and named according to the base substitution present in the fourth repeat (**A**) or the second and fourth repeats (**E**) at the G1, G2 or G3 positions. Complete sequences shown in Supplementary Tables 1 and 2. ELISA binding curves for telomeric constructs containing a single (**B**) or double (**F**) base substitution. BG4 binding is represented by absorbance at 450nm vs. the BG4 concentration (nM). (**C**) and (**G**) apparent dissociation constants (K_d_) were calculated from the nonlinear regression curves shown in (**B**) and (**F**), respectively. Data are mean ± SD from independent experiments; ordinary one-way ANOVA. (**D**) Quantification of SiMPull counts of BG4 binding events with telomeric constructs containing a single base substitution in the fourth repeat. TELO4 data from Figure 1E. Data are mean ± SD from three independent experiments; ordinary one-way ANOVA. *P < 0.1, **P< 0.01, ****P< 0.0001.

Next, we examined how base substitutions in the telomeric sequence affect BG4 binding since mutations alter GQ folding and properties (26) (Figure 3A). As a control, we used a construct with a thymidine to cytidine substitution (T2C) in the ‘TTA’ loop sequence between the third and fourth guanine runs, since substitutions in the loops have a minor effect on telomeric GQ stability (23). As expected, BG4 showed similar affinity for the T2C construct, with an apparent *K*_d_ of 5.2 ± 1.5 nM compared to TELO4 with a value of 5.7 ± 0.7 nM (Figure 3B, C). However, substitutions that alter guanines generally reduced BG4’s affinity, likely due to the loss of a G-quartet to form a stable GQ. Substitutions at both the first (G1) and second (G2) guanine in the fourth repeat yielded apparent K_d_ values for BG4 binding ranging from 7.3 ± 0.9 nM for the G1T substrate to 13.9 ± 4.1 nM for the G2C substrate (Figures 3A–C). We showed previously that altering G2, which participates in the central G-quartet, has a greater impact on GQ dynamics and stability than changing the first or third Gs where a two-tier GQ is still possible (26). Consistent with this study, substitutions at G2 increased the apparent *K*_d_ more than changes at the G1 position, which was significantly different for the G to C substitution. BG4 also has higher affinity for substrates with G to A or G to T substitutions than for substrates with G to C substitutions (Figure 3A–C). This contrasts our previous finding of GQ instability in the order of C < T < A substitutions for G in telomeric GQ by smFRET (26). However, BG4 binds all of the substrates containing G base substitutions with apparent *K*_d_ values greater than the negative controls shown in Supplementary Figure 1. These data suggest BG4 can bind partially folded telomeric GQ.

Next, we examined how base substitutions in the telomeric constructs impact BG4 binding at the single molecule level by SiMPull. We observed similar patterns in BG4 binding to constructs with single base substitutions in the fourth repeat by the two assays, except the substitutions showed a greater reduction in BG4 affinity by SiMPull analysis, compared to ELISA (Figures 3C compared to 3D). BG4 binding was significantly reduced for all base substitution constructs tested, from 328 ± 53 counts for TELO4 to between 29 ± 7 (G2T) and 111 ± 14 (G1C). As for ELISA, the extent of affinity reduction depended on the base substitution position, with all three substrates containing substitutions at G2 showing reduced binding compared to the equivalent substitution at G1 or G3 (Figure 3D). G to C substitutions were the least disruptive to BG4 binding by SiMPull, which contrasted with the ELISA results. Collectively, these data show that base substitutions that disrupt GQ stability reduce, but do not prevent, BG4 binding.

To further impair GQ folding, we designed a second set of constructs containing substitutions in both the second and fourth repeats (Figure 3E). As with the 7dzG substitutions (Figure 2), all the double-substitution constructs show greatly reduced BG4 binding, compared to the single-substitution counterparts, with most of the apparent *K*_d_ values near the maximal BG4 concentration used in the assay (40 nM) (Figures 3F, G). Again, G to T substitution at the first position affected BG4 binding the least, only increasing the apparent *K*_d_ to 11.0 ± 2.0 nM for the 2xG1T substrate. The G to C substitutions were highly disruptive, yielding an apparent K_d_ of 24.0 ± 2.6 nM for the 2xG1C substrate and exceeding 40 nM for the 2xG2C. In contrast with the results for the single-substitutions, the double G to A substitutions disrupted BG4 binding the most, and a hyperbolic binding curve could not be fit for the 2xG2A construct. BG4 binding to the substrates with multiple substitutions is lower than for substrates with single substitutions, providing additional evidence that BG4 can bind partially folded GQ substrates.

### BG4 can bind to telomeric GQ harboring damaged bases

**Figure 4.**
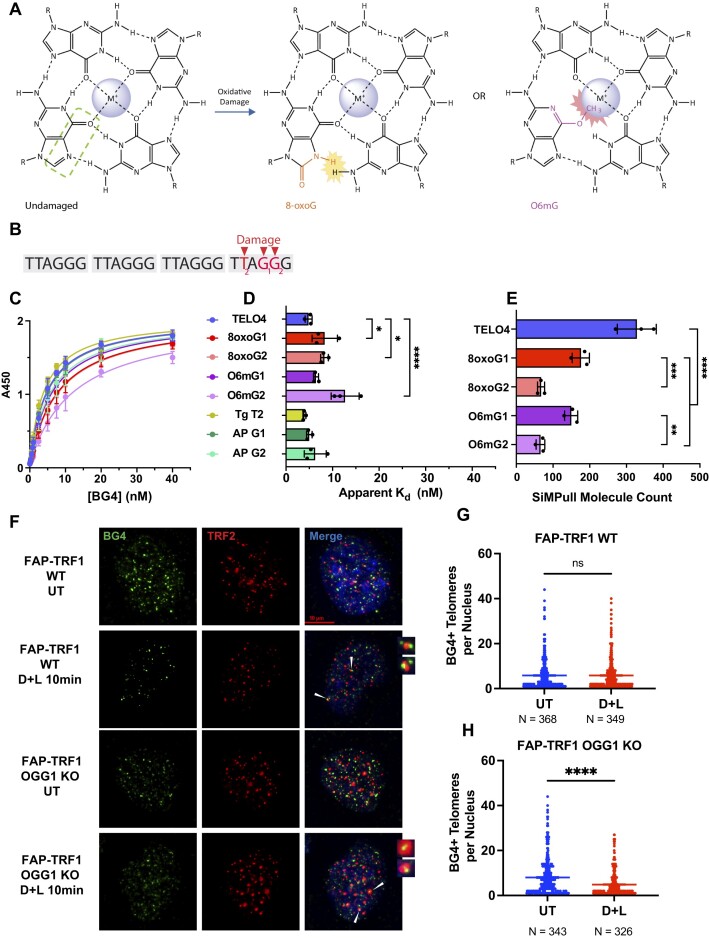
BG4 binds GQ harboring guanine lesions although with reduced affinity. (**A**) Chemical structure of 8-oxoG or O6mG in a G-quartet demonstrating loss of Hoogsteen bonding with adjacent guanine and steric clashes. (**B**) Constructs are based on TELO4 and named according to the damaged base (red) present in the fourth telomeric repeat of the T2, G1 or G2 position. Complete sequences shown in Supplementary Tables 1 and 2. (**C**) ELISA binding curves for telomeric oligonucleotides containing damaged guanine base derivatives. BG4 binding is represented by absorbance at 450nm vs. the BG4 concentration. (**D**) Apparent *K*_d_ values were calculated from the nonlinear regression curves shown in (C). Data are mean ± SD from 3 independent experiments; ordinary one-way ANOVA. (**E**) Quantification of SiMPull counts of BG4 binding events with telomeric constructs containing a guanine lesion in the fourth repeat. TELO4 data from Figure 1E. Data are mean ± SD from three independent experiments; ordinary one-way ANOVA. (**F**) Representative IF images showing BG4 (green) colocalization with telomeric protein TRF2 (red) in FAP-TRF1 U2OS WT or OGG1 KO (clone 13) cells untreated (UT) or after telomeric 8oxoG induction with 10 min MG2I dye and 660 nM light (DL) exposure. White arrowheads point to BG4+ telomeres. (G, H) Quantification of the number of BG4 foci colocalized with telomeres per nuclei analyzed for WT (**G**) and OGG1 KO cells (**H**); each dot represents a nucleus. Data are mean ± S.D; unpaired *t* test with Welsh's correction comparisons test. Not significant = ns or no symbol, *P < 0.1, **P< 0.01, ***P< 0.001, ****P< 0.0001.

Next, we asked whether damaged bases known to alter telomeric GQ properties, also alter BG4 affinity (Figure 4A). We showed by smFRET that a thymine glycol (Tg) lesion at the second T in the fourth repeat (T2Tg) does not reduce GQ stability, but rather alters the distribution of telomeric GQ conformations (26). However, BG4 affinity for the T2Tg construct was similar to the unmodified TELO4, with a *K*_d_ of 4.0 ± 0.3 nM (Figures 4B–D). In contrast to Tg, smFRET studies showed that 8-oxoguanine (8-oxoG) and O6-methylguanine (O6mG) damage disrupt GQ stability, but allow for partial GQ folding (26). These lesions each disrupt the Hoogsteen binding face and introduce steric clashes that impact GQ folding (Figure 4A), however, telomeric GQs with an 8-oxoG retain the hybrid conformation (25). 8-oxoG or O6mG substitutions for the first or second G in the fourth repeat reduced BG4 binding as expected, but affinity was surprisingly greater than for non-GQ negative controls (Supplementary Figure 1 and Figures 4B, C). Similar to results with substrates containing base mutations, damage at G2 reduced BG4 binding more than damage at G1, except for the 8-oxoG lesion (Figures 4B–D). O6mG at G2 was most disruptive to BG4 binding, with an apparent *K*_d_ of 13 ± 3.0 nM, compared to 8-oxoG in the second position with an apparent *K*_d_ of 8.2 ± 0.8 nM. An abasic lesion at G1 or G2 did not reduce BG4 binding relative to TELO4, yielding apparent *K*_d_ values of 5.0 ± 0.6 and 6.3 ± 2.4 nM, respectively, consistent with reports that telomeric GQ harboring an abasic analog can form stable GQ (47). As for constructs harboring a base substitution, the 8-oxoG and O6mG lesions showed a greater impact on BG4 binding by SiMPull assay, than by ELISA (Figure 4E). Modification of the G2 disrupted BG4 binding significantly more than modification at either G1 (Figure 4E). BG4 bound an average of 176 ± 23 molecules containing an 8-oxoG at G1, compared to 67 ± 10 molecules with an 8-oxoG at G2. Interestingly, the base substitutions led to a greater reduction in relative BG4 binding, compared to the damaged bases, as measured by both ELISA and SiMPull (Figures 3B–D and 4C–E). Collectively, these data indicate that BG4 can bind to partially-folded GQ conformations containing guanine lesions.

Next, we tested whether 8-oxoG damage alters BG4 staining by immunofluorescence. The production of 8-oxoG at telomeres using the FAP-MG2I chemoptogenetic system (33) did not significantly reduce telomeric foci colocalized with BG4 in wild type cells, while repair deficient OGG1 knock out cells showed a decrease from 8 to 5 average BG4+ telomeres/nucleus (Figure 4F–H and Supplementary Figure 3). Damage in wild type cells may not have reached the threshold to significantly reduce BG4 binding. To test how genome-wide damage impacts BG4 staining, we treated cells with oxidant KBrO_3_, which primarily produces 8-oxoG based on mutational signature analysis (48). KBrO_3_ treatment reduced the overall number of BG4 foci per nucleus up to 1.4-fold in wild type cells, and up to 3.3-fold in OGG1 knock out cells (Supplementary Figure 3). Interestingly, the OGG1 knock out clone showed higher basal BG4 staining, which may be due to replication stress from unrepaired lesions and increased single stranded DNA that can fold into GQ. These data agree with biochemical results that 8-oxoG reduces BG4 affinity for GQ, and suggest a damage threshold is required to reduce BG4 staining by IF.

### BG4 stabilizes GQ harboring disruptive guanine modifications

**Figure 5.**
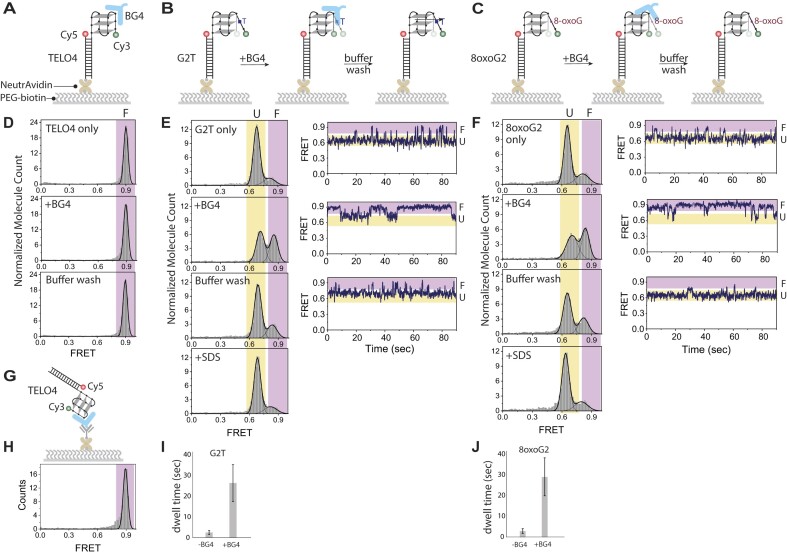
BG4 addition promotes GQ folding of telomeric constructs containing a destabilizing G substitution. Schematics for biotinylated FRET constructs TELO4 (**A**), G2T (**B**), or 8oxoG2 (**C**) tethered to neutravidin coated slides, followed by 150 nM BG4 addition and then buffer washes. Construct sequences shown in Supplementary Table 2. Histograms of FRET efficiency frequencies observed TELO4 (**D**), G2T (**E**), and 8oxoG2 (**F**) before or after BG4 addition, and sequential buffer wash and SDS addition as indicated. Representative single-molecule traces show FRET efficiencies captured over 90 s. (**G**) Schematic of SiMPull assay for FRET non-biotinylated FRET TELO4 construct binding to BG4 attached to the slide via FLAG antibody. (**H**) Histogram of FRET efficiency after adding non-biotinylated TELO4 FRET construct. (**I, J**) Dwell times for the high-FRET state of G2T and 8oxoG2 constructs in the presence and absence of BG4 from panels E and F. Colors indicate conformations for a range of FRET efficiencies: orange for partially unfolded GQ (marked with U) and blue for folded GQ (marked with F).

We used smFRET to determine whether molecules bound by BG4 remain in a GQ folded state. The smFRET constructs contained a donor dye (Cy3) at the 3′ end of single stranded telomeric DNA with the acceptor dye (Cy5) at the 5′ end of complementary stem containing 3′ biotin for surface immobilization (Figure 5A-C). Since GQ folding yields high FRET between the Cy3 and Cy5 pair (26), we recorded FRET efficiencies to monitor GQ folding and stability before and after adding BG4 to the imaging chamber, and after washes post BG4 addition. The TELO4 constructs showed a high FRET peak at 0.9 indicative of GQ folding that remained unaltered upon BG4 addition and following buffer washes (Figure 5D). The reverse experiment in which we bound BG4 to the surface instead and added the non-biotinylated TELO4 FRET construct, confirmed that BG4 binding did not alter the FRET efficiency (Figures 5G, H). To determine whether BG4 could promote GQ folding of constructs harboring a destabilizing G substitution, we tested biotinylated telomeric constructs bound to the surface that contained a G to T (G2T) or an 8-oxoG at G2 (8oxoG2) in the fourth repeat. Prior to BG4 addition, both constructs showed a major FRET peak at about 0.65 indicative of a partially folded GQ, and a minor FRET peak near 0.85 indicative of a folded GQ (Figures 5B, C and E, F). The smFRET traces showed dynamics with prolong partially folded mid-FRET state (∼0.65) with very transient movements to the high-FRET folded state. This dynamic behavior is due to the base substitution or damaged guanine destabilizing the GQ structure and is consistent with previous findings (26). Interestingly, BG4 addition (150 nM) increased the high FRET population to nearly 50% for both the G2T and 8oxoG2 constructs, and increased prolonged high FRET folded state, compared to the unbound constructs (Figures 5E, F). The dwell time of the high-FRET state increased over 10-fold for both G2T and 8oxoG2 constructs in the presence of BG4 (Figures 5I, J). Buffer washes dramatically reduced the high FRET peak, and SDS addition to denature BG4 restored the FRET histograms to those obtained prior to BG4 addition. These washes confirm that BG4 binding was responsible for the shift to high FRET states. Interestingly, the induced high FRET peak was greater upon BG4 addition and after buffer washes for the 8oxoG2 construct, compared to the G2T construct, consistent with higher affinity for the former (Figures 3 and 4). These data suggest that BG4 binding to telomeric constructs harboring disrupting G substitutions can promote GQ folding and stability, but that BG4 can be removed with extensive washes.

### BG4 binds to a G-quadruplex with 1:1 stoichiometry

**Figure 6.**
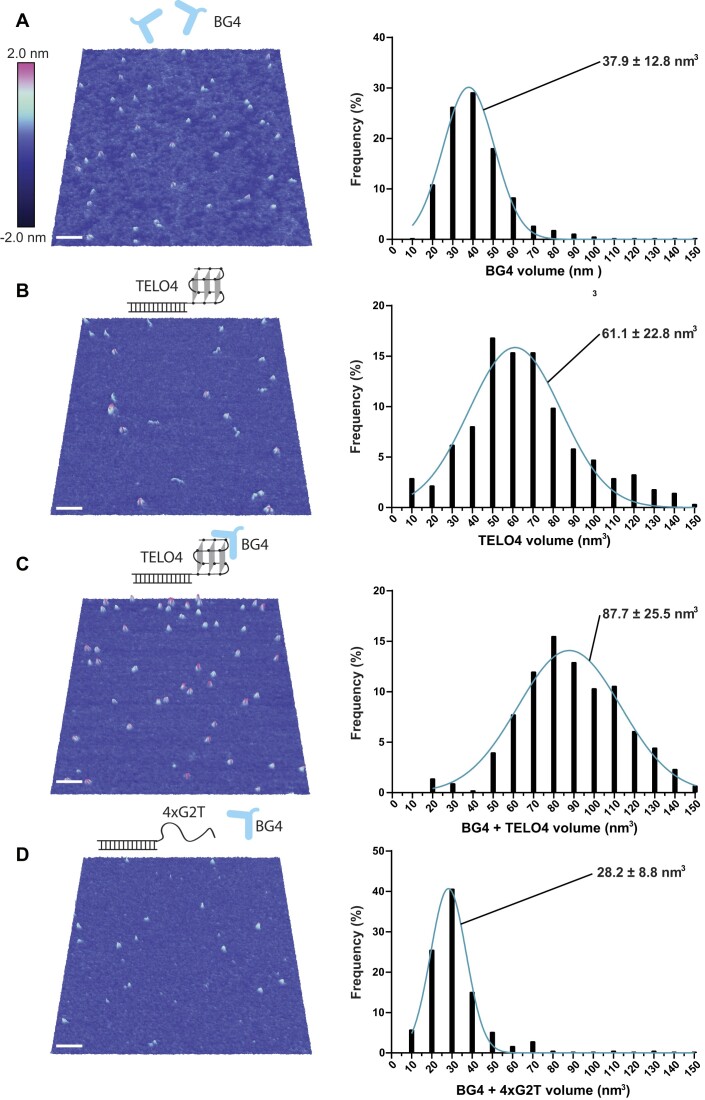
BG4 binds the telomeric GQ with 1:1 stoichiometry. Representative three-dimensional AFM images and volume histograms for (**A**) 8 nM BG4, *n* = 696 particles, (**B**) 250 mM TELO4, *n* = 273 particles, (**C**) 4 nM BG4 reacted with 1 nM TELO4, *n* = 425 particles, and (**D**) 4 nM BG4 reacted with 1 nM 4xG2T, *n* = 173 particles. Gaussian distributions fit to the histograms are shown with mean ± S.D. White scale bar indicates 50 nm, and color scale at top represents height above surface. Each experiment was repeated at least 2–4 times and consisted of multiple depositions per experiment.

To determine the stoichiometry of BG4 binding to telomeric GQ, we used single molecule AFM imaging. The deposition of 8 nM BG4 alone revealed a nearly homogeneous collection of small distinct particles lacking large aggregates or contaminating DNA (Figure 6A). Histograms of particle volume reveal a tight Gaussian distribution corresponding to a mean BG4 particle volume of 37.9 ± 12.8 nm^3^ (Figure 6B). AFM volumes of proteins are generally proportional to their molecular weights (49,50). Using a calibration curve (Supplementary Figure 4) (51), we calculated a mean BG4 particle molecular weight of 32.1 ± 12.0 kDa, which agrees well with the 32.7 kDa MW based on the amino acid sequence. Monomer-sized particles are roughly 6-fold more prevalent than particles with weights corresponding to a dimer, demonstrating that BG4 is primarily monomeric. In agreement, purified BG4 is monomeric in solution based on size exclusion chromatography (Supplementary Figure 1A).

To determine if AFM could distinguish between BG4-bound telomeric GQ and unbound GQ, we imaged DNA constructs consisting of a 34-bp duplex stem and a 3′ single-strand overhang of four TTAGGG repeats as described previously (41). Depositions of this construct alone showed particles with a mean volume of 61.1 ± 22.8 nm^3^, notably larger than BG4 particles (Figure 6B). Depositions of 4 nM GQ substrate incubated with 1 nM BG4 for 30 min, showed particles with a mean volume of 87.7 ± 25.5 nm^3^ (Figure 6C), which was roughly equal to the sum of the volumes for one BG4 and one GQ substrate. These results suggest that the particles contain a 1:1 ratio of BG4 bound to GQ, and that BG4 binds as a monomer. As a negative control, we observed only small particles with a mean volume of 28.2 ± 8.8 nm^3^ when BG4 was mixed with a non-GQ substrate in which G2 of each repeat was mutated to T (4XG2T). We confirmed by AFM previously that this substrate does not form a GQ (41). The smaller volumes more closely resemble particles of unbound BG4 alone (Figures 6A and D). These results provide further evidence that BG4 binding is specific to GQ structures, and reveal that BG4 binds to GQ as a monomer with 1:1 stoichiometry.

## Discussion

The G-quadruplex antibody BG4 is commonly used to detect GQ structures in cells and at telomeres by immunofluorescence (IF) or chromatin immunoprecipitation (ChIP). To determine whether base mutations and damage at telomeres that disrupt GQ folding would reduce BG4 binding, we characterized BG4’s affinity for various telomeric substrates containing site-specific base modifications. Both ELISA and single-molecule pull down revealed that BG4 has high affinity for telomeric GQ containing four or more TTAGGG repeats. Unexpectedly, we found BG4 also binds single molecules of 3 repeat constructs, although with reduced affinity of about 2–3-fold. Equally surprising, we found BG4 can bind constructs containing a guanine base substitution or lesion that disrupts a Hoogsteen bond, although also with reduced affinity. However, multiple guanine substitutions that unfold the GQ by disrupting more than one quartet nearly abolished BG4 binding. Single molecule FRET assays to monitor GQ folding revealed that BG4 binding promotes folding of telomeric GQs harboring a single G to T or 8-oxoguanine substitution. Finally, we demonstrate that BG4 binds to telomeric GQ substrates with 1:1 stoichiometry. Our studies reveal that BG4 can recognize partially folded telomeric GQ structures, and promote telomeric GQ stability.

**Figure 7.**
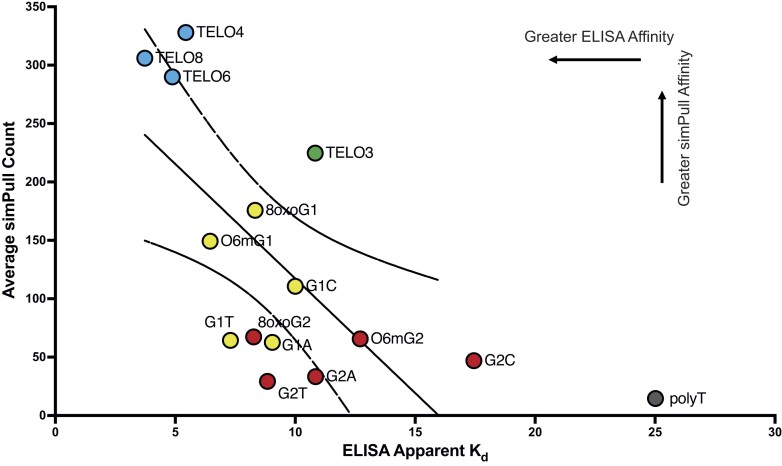
Correlation between BG4 affinities for various constructs determined by SiMPull and ELISA. Average SiMPull counts for selected constructs plotted vs. the average calculated apparent *K*_d_ determined by ELISA. Telomeric constructs that differ by repeat number are shown in blue, four repeat constructs with a modified G1 base are indicated in yellow, four repeat constructs with a modified G2 base are indicated in purple, and the polythymidine construct is indicated in dark grey. Pearson correlation coefficient (*r*) = –0.642, *P* = 0.0132. The polythymidine construct was omitted from statistical analysis.

Our results show good agreement between the bulk and single molecule binding assays for BG4 affinity to telomeric substrates. While single molecule assays offer the advantage of providing information related to structure conformation and dynamics, ELISA is widely used for testing antibody affinity and specificity. Both assays lack chemical fixation steps, so dynamics of GQ folding are permitted. Plotting the SiMPull counts versus the apparent *K*_d_ values from ELISA confirm good correlation between the assays, and reveals constructs with similar GQ stability cluster together (Figure 7). The stable GQs formed by constructs with 4–8 telomeric repeats show high affinity, and the construct with three repeats shows intermediate binding, by both methods. The substrates containing DNA lesions or base substitutions at either guanine position 1 or position 2 also cluster, with position 2 modifications generally showing the weakest binding in both ELISA and SiMPull. The poly-thymidine control is an outlier since BG4 showed intermediate binding to T_25_ by ELISA, but very little binding to T_24_ by SiMPull. The differences may be explained by the experimental setup. In ELISA the pre-folded biotinylated constructs are bound to the plates, whereas in SiMPull the BG4 protein is attached to the slide via a FLAG antibody. Previous studies with the GQ antibody 1H6 showed moderate affinity for (T)_15_ by ELISA, but poor binding by the solution-based microscale thermophoresis (52). The authors suggested the restricted movement of the poly(T) substrate in the ELISA platform may enable 1H6 interaction. Similarly, greater restriction of the poly-T attached to the ELISA plate, compared to addition to the SiMPull imaging chamber, may facilitate BG4 interaction. Never-the-less, good agreement between the ELISA and SiMPull assays for measuring BG4 affinity for telomeric GQ constructs, suggests that both assays are suitable for testing BG4 interaction with various substrates. Since ELISA is a bulk biochemical assay, we propose SiMPull may provide a better guide for cellular assays aimed a detecting BG4 binding to individual sites of GQ folding in the genome.

Our results agree well with reported BG4 binding to similar substrates previously analyzed by ELISA. Our calculated apparent K_d_ values for TELO4 and KIT1 GQ substrates are similar to those reported by Biffi *et al.* (31), although slightly higher at 5.0 and 6.5 nM, compared to 1.1 and 1.5 nM, respectively. This may be due to differences in DNA concentrations and ELISA buffer composition. Our extension of this analysis showing BG4 retains high affinity for telomeric GQ containing more than four repeats increases confidence that BG4 recognizes telomeric GQ folds in cells, since multiple conformations can arise in the context of telomeres including GQs with loops containing full repeats. This previous study also reported BG4 binding to a (TTAGGG)_3_ construct but interpreted this as evidence that BG4 binds to bi-molecular GQ formed between two molecules, since TELO3 cannot form an intramolecular GQ (31). However, we argue bi-molecular GQ formation in our experimental setup is unlikely since both ELISA and SiMPull used low DNA concentrations (10 nM), and our single molecule work showed the majority of bound substrates are unimolecular. Rather, we propose BG4 binds partially folded GQ such as G-hairpin or G-triplex structures formed by Hoogsteen bonding, although they are less stable since quartets cannot form. Consistent with this, we reported by smFRET that TELO3 forms FRET histogram peaks centered at 0.65 and 0.8, indicative of a compact structure (37).

Further support for BG4 binding to partially folded, but destabilized telomeric GQ, derives from studies that suggest GQs fold and unfold in a stepwise manner with intermediates likely consisting of G-hairpin and G-triplex structures forming between fewer than four G-tracts (53–56). While the conformational diversity in these folding pathways remains to be fully understood, circular dichroism and molecular dynamic simulation experiments predict the presence of partially-folded Hoogsteen duplexes and triplexes before a fully stable GQ is formed (57–59). Such structures may also form when one of the G runs in the (TTAGGG)_4_ construct is altered thereby preventing a Hoogsteen bond and quartet. For example, BG4 shows similar affinity for TELO3 as for TELO4 harboring a single 7dzG substitution. In addition, a G substitution in two repeats more strongly reduces BG4 affinity than a substitution in one repeat in which the constructs retain three G runs. Previous smFRET experiments of telomeric GQ with single base substitutions, 8-oxoG, or O6mG at the G1, G2 or G3 position also provide evidence for these GQ intermediates. FRET histograms of constructs with modifications at the G1 or G3 position show peaks between 0.8 and 0.9, while modifications at the G2 position show mid-FRET peaks ranging from 0.5 to 0.7 (26). In agreement, we observed substitution at the G2 reduced BG4 binding greater than substitution at the G1 or G3 position by SiMPull. The mid-FRET peak corresponds to a partially folded state which may arise from a G-triplex as the FRET efficiency is higher than for the unstructured poly-T_24_ DNA (26). Therefore, our results suggest that BG4 may bind G-triplex as well as partially-folded GQ, represented by the mid- to high-FRET population of molecules.

We propose our finding that BG4 binds to GQ constructs containing partially folded or intermediate GQ states, is likely explained by BG4’s ability to stabilize these GQ constructs upon binding. In support of this, smFRET assays show substituting the 2nd G with a T or 8-oxoG in the telomeric repeat shifts molecules from a high FRET static behavior, indicative of stably folded GQ, to mid-FRET dynamic behavior that fluctuates within the mid-FRET range (23,26) (Figures 5D–F). BG4 addition not only greatly increased the number of molecules exhibiting high FRET, but also increased the duration of molecules residing in the high FRET states. These results strongly suggest that BG4 promotes GQ folding of destabilized GQ constructs through its binding action. In these experiments, the BG4 concentration was 150 nM, roughly 20 times the apparent *K*_d_ calculated for BG4’s binding to these substrates. Consistent with our results, a previous study reported BG4 increased the formation rate and persistence of a (GGGCTA)_4_ GQ (60). Furthermore, APE1 repair protein binding to GQ constructs harboring an abasic site promotes GQ folding (61). Our results also suggest that BG4 expression in cells might promote the stabilization of GQ structures, similar to that reported for small molecule GQ ligands (17). This suggests BG4 expression in cells may interfere with telomeric replication or telomerase activity by stabilizing telomeric GQ.

What we learned about BG4’s affinity for various telomeric GQ can inform about its use for GQ detection at telomeres in cells by IF or ChIP. First, we found BG4 binds to GQ structures with 1:1 stoichiometry, similar to many GQ ligands including PDS (62), NMM (63), PhenDC3 (64) and telomestatin (65). Ligand binding is primarily by interactions between their ring structures and the planar distal G-quartet. Determining the BG4 epitope will require high resolution structural studies, but the binding stoichiometry has implications for IF. Detecting a single GQ in a promoter, for example, may be challenging, and previous reports of BG4 staining at telomeres may represent a cluster of GQ structures or may be underestimated (31,32,66). Methods that enhance sensitivity and signal detection such as the proximity ligation assay may improve BG4 detection of individual GQ structures in cells. Second, the ability of BG4 to bind (TTAGGG)_3_ folds suggests BG4 telomere localization in cells could be due to G-triplex binding. However, the majority of telomeres lack BG4 foci by IF (31), suggesting G-triplexes are infrequent or unstable in cells and/or washes disrupt BG4 binding to (TTAGGG)_3_ folds given the reduced affinity compared to GQ. Third, BG4 binding to partial or intermediate telomeric GQ folds suggests that using BG4 to detect reduced GQ formation in cells due to destabilizing factors may be challenging. However, our result that smFRET histograms for the telomeric GQ containing an 8-oxoG or T substitutions return to BG4-unbound states after washing, suggests that extra washes in IF or ChIP may disrupt BG4 binding to the less stable GQ. Consistent with this, we observed telomere specific 8-oxoG damage decreased BG4 staining at telomeres by IF in repair deficient cells. Nevertheless, BG4 binding and stabilization of partially folded telomeric GQs should be considered when interpreting experimental results. For example, ChIP-Seq studies revealing overlapping regions pulled down by BG4 and antibodies against proteins that bind abasic sites (APE1) and 8-oxoG (OGG1), suggest that BG4 may bind to GQs containing these lesions (61). In summary, our studies reveal that BG4 not only shows flexibility in binding GQ of various conformations, but can also bind to partially-folded GQ telomeric structures containing destabilizing G base substitutions.

## Supplementary Material

gkad1209_Supplemental_File

## Data Availability

All raw data is available and can be released upon request from the corresponding author, plo4@pitt.edu.
