# Supplementary material for: BG4 antibody can recognize telomeric G-quadruplexes harboring destabilizing base modifications and lesions
Source: Nucleic Acids Res. 2023 Dec 28;52(4):1763–78. doi: 10.1093/nar/gkad1209 (PMC10939409; doi:10.1093/nar/gkad1209)
Supplement: gkad1209_Supplemental_File [file gkad1209_supplemental_file.pdf]

Supplementary Data

BG4 antibody can recognize telomeric G-quadruplexes harboring destabilizing base modifications and lesions

Samuel Johnson[1,2,3#], Tapas Paul[5,6#], Samantha L. Sanford[1,2#], Brittani L Schnable[2,3], Ariana Detwiler[1,2], Sanjana A. Thosar[1,2], Bennett Van Houten[2,3,4], Sua Myong[5,6], Patricia L. Opresko[1,2,3,4*]

[1]Department of Environmental and Occupational Health, University of Pittsburgh School of Public Health, Pittsburgh, PA, USA

[2]UPMC Hillman Cancer Center, Pittsburgh, PA, USA

[3]Molecular Biophysics and Structural Biology Graduate Program, University of Pittsburgh, PA, USA

[4]Department of Pharmacology and Chemical Biology, University of Pittsburgh School of Medicine, PA, USA

[5]Department of Biophysics, Johns Hopkins University, Baltimore, MD 21218, USA

[6]Program in Cellular and Molecular Medicine, Boston Children's Hospital, Boston, MA, USA.

[#]Authors contributed equally

*To whom correspondence should be addressed. plo4@pitt.edu.

Supplementary Table 1
Supplementary Table 2
Supplementary Table 3
Supplementary Figure 1
Supplementary Figure 2
Supplementary Figure 3
Supplementary Figure 4

Supplementary Table 1. Oligonucleotides used in ELISA experiments

Name	Sequence
18-mer	5'/ GCC TCG CTG CCG TCG CCA/ 3'
TELO4	5' Bio/ TGG CGA CGG CAG CGA GGC (TTAGGG)$_4$ /3'
KIT1	5' Bio/ TGG CGA CGG CAG CGA GGC AGG GAG GGC GCT GGG AGG AGG G /3'
T25	5' Bio/ TGG CGA CGG CAG CGA GGC (T)$_{25}$ /3'
ssDNA	5' Bio/ TGG CGA CGG CAG CGA GGC ATA GTG CGT GGG CG/ 3'
dsDNA	5' Bio/ TGG CGA CGG CAG CGA GGC ATA GTG CGT GGG CG/ 3'<br>3'/ ACC GCT GCC GTC GCT CCG TAT CAC GCA CCC GC/ 5'
32-mer	5' / CG CCC ACG CAC TAT GCC TCG CTG CCG TCG CCA/ 3'
TELO8	5' Bio/ TGG CGA CGG CAG CGA GGC (TTAGGG)$_8$/ 3'
TELO7	5' Bio/ TGG CGA CGG CAG CGA GGC (TTAGGG)$_7$/ 3'
TELO6	5' Bio/ TGG CGA CGG CAG CGA GGC (TTAGGG)$_6$/ 3'
TELO5	5' Bio/ TGG CGA CGG CAG CGA GGC (TTAGGG)$_5$/ 3'
TELO3	5' Bio/ TGG CGA CGG CAG CGA GGC (TTAGGG)$_3$/ 3'
TELO4 7dzG	5' Bio/ TGG CGA CGG CAG CGA GGC (TTAGGG)$_3$ TTA G(7dzG)G/ 3'
TELO4 2x7dzG	5' Bio/ TGG CGA CGG CAG CGA GGC TTA GGG TTA G(7dzG)G TTA GGG TTA G(7dzG)G/ 3'
TELO3 7dzG	5' Bio/ TGG CGA CGG CAG CGA GGC TTAGGGTTAGGGTTAG(7dzG)G/ 3'
T2C	5' Bio/ TGG CGA CGG CAG CGA GGC (TTAGGG)$_3$ TCA GGG/3'
G1T	5' Bio/ TGG CGA CGG CAG CGA GGC (TTAGGG)$_3$ TTA TGG/ 3'
G2T	5' Bio/ TGG CGA CGG CAG CGA GGC (TTAGGG)$_3$ TTA GTG/ 3'
G1A	5' Bio/ TGG CGA CGG CAG CGA GGC (TTAGGG)$_3$ TTA AGG/ 3'
G2A	5' Bio/ TGG CGA CGG CAG CGA GGC (TTAGGG)$_3$ TTA GAG/ 3'
G1C	5' Bio/ TGG CGA CGG CAG CGA GGC (TTAGGG)$_3$ TTA CGG/ 3'
G2C	5' Bio/ TGG CGA CGG CAG CGA GGC (TTAGGG)$_3$ TTA GCG/ 3'
2xG1T	5' Bio/ TGG CGA CGG CAG CGA GGC TTA GGG TTA TGG TTA GGG TTA TGG/ 3'
2xG2T	5' Bio/ TGG CGA CGG CAG CGA GGC TTA GGG TTA GTG TTA GGG TTA GTG/ 3'
2xG1A	5' Bio/ TGG CGA CGG CAG CGA GGC TTA GGG TTA AGG TTA GGG TTA AGG/ 3'
2xG2A	5' Bio/ TGG CGA CGG CAG CGA GGC TTA GGG TTA GAG TTA GGG TTA GAG/ 3'
2xG1C	5' Bio/ TGG CGA CGG CAG CGA GGC TTA GGG TTA CGG TTA GGG TTA CGG/ 3'
2xG2C	5' Bio/ TGG CGA CGG CAG CGA GGC TTA GGG TTA GCG TTA GGG TTA GCG/ 3'
Tg T2	5' Bio/ TGG CGA CGG CAG CGA GGC (TTAGGG)$_3$ T(Tg)A GGG/ 3'
AP G1	5' Bio/ TGG CGA CGG CAG CGA GGC (TTAGGG)$_3$ TTA (AP)GG/ 3'
AP G2	5' Bio/ TGG CGA CGG CAG CGA GGC (TTAGGG)$_3$ TTA G(AP)G/ 3'
8oxoG1	5' Bio/ TGG CGA CGG CAG CGA GGC (TTAGGG)$_3$ TTA (8oxoG)GG/ 3'
8oxoG2	5' Bio/ TGG CGA CGG CAG CGA GGC (TTAGGG)$_3$ TTA G(8oxoG)G/ 3'
O6meG1	5' Bio/ TGG CGA CGG CAG CGA GGC (TTAGGG)$_3$ TTA (O6mG)GG/ 3'
O6meG2	5' Bio/ TGG CGA CGG CAG CGA GGC (TTAGGG)$_3$ TTA G(O6mG)G/ 3'

Bio = biotin. Base substitutions are underlined. 7dzG = 8-aza-7-deazaguanine, Tg = thymine glycol ($C_5H_8N_2O_4$), AP = apurinic/apyrimidinic analog tetrahydrofuran (C_4H_8O), 8oxoG = 8-oxoguanine, and O6mG = O6-methylguanine. All biotinylated oligonucleotides were annealed to the 18-mer oligonucleotide to generate a duplex stem, except the dsDNA was annealed to the 32-mer.

Supplementary Table 2. Oligonucleotides used in SiMPull and FRET experiments

Name	Sequence
TELO3	5'/ TGG CGA CGG CAG CGA GGC (TTAGGG)$_3$ /3'Cy3/
TELO4	5'/ TGG CGA CGG CAG CGA GGC (TTAGGG)$_4$ /3'Cy3/
TELO6	5'/ TGG CGA CGG CAG CGA GGC (TTAGGG)$_6$ /3'Cy3/
TELO8	5'/ TGG CGA CGG CAG CGA GGC (TTAGGG)$_8$ /3'Cy3/
T$_{24}$	5'/ TGG CGA CGG CAG CGA GGC (T)$_{24}$ /3'Cy3
G1T	5'/ TGG CGA CGG CAG CGA GGC (TTAGGG)$_3$ TTA TGG/ 3'Cy3/
G2T	5'/ TGG CGA CGG CAG CGA GGC (TTAGGG)$_3$ TTA GTG /3'Cy3/
G3T	5'/ TGG CGA CGG CAG CGA GGC (TTAGGG)$_3$ TTA GGT /3'Cy3/
G1A	5'/ TGG CGA CGG CAG CGA GGC (TTAGGG)$_3$ TTA AGG /3'Cy3/
G2A	5'/ TGG CGA CGG CAG CGA GGC (TTAGGG)$_3$ TTA GAG /3'Cy3/
G3A	5'/ TGG CGA CGG CAG CGA GGC (TTAGGG)$_3$ TTA GGA /3'Cy3/
G1C	5'/ TGG CGA CGG CAG CGA GGC (TTAGGG)$_3$ TTA CGG /3'Cy3/
G2C	5'/ TGG CGA CGG CAG CGA GGC (TTAGGG)$_3$ TTA GCG /3'Cy3/
G3C	5'/ TGG CGA CGG CAG CGA GGC (TTAGGG)$_3$ TTA GGC /3'Cy3/
8oxoG1	5'/ TGG CGA CGG CAG CGA GGC (TTAGGG)$_3$ TTA (8oxoG)GG /3'Cy3/
8oxoG2	5'/ TGG CGA CGG CAG CGA GGC (TTAGGG)$_3$ TTA G(8oxoG)G /3'Cy3/
8oxoG3	5'/ TGG CGA CGG CAG CGA GGC (TTAGGG)$_3$ TTA GG(8oxoG) /3'Cy3/
O6mG1	5'/ TGG CGA CGG CAG CGA GGC (TTAGGG)$_3$ TTA (O6mG)GG /3'Cy3/
O6mG2	5'/ TGG CGA CGG CAG CGA GGC (TTAGGG)$_3$ TTA G(O6mG)G /3'Cy3/
O6mG3	5'/ TGG CGA CGG CAG CGA GGC (TTAGGG)$_3$ TTA GG(O6mG) /3'Cy3/
Cy5-18mer-Bio	5' /Cy5/ GCC TCG CTG CCG TCG CCA /Bio/ 3'
Cy5-18mer	5' /Cy5/ GCC TCG CTG CCG TCG CCA 3'

Bio = biotin. Base substitutions are underlined. 8oxoG = 8-oxoguanine, and O6mG = O6-methylguanine. Oligonucleotides were annealed to the 18-mer oligonucleotide for SiMPull (Supplementary Table 1, Figures 1, 3, 4), the Cy5-18mer-Bio for smFRET (Figures 5A-F and 5I-J), or the Cy5-18-mer (TELO4, Figure 5G-H). Cy3 and Cy5 dyes were attached as described in Materials and Methods.

Supplementary Table 3. Oligonucleotide constructs used in AFM experiments

Name	Annealed Sequence
TELO4	5’-AGGTATCAGCATAATGGCCACGGTGCGTACTGCG(TTAGGG)$_4$-3’<br>3’-TCCATAGTCGTATTACCGGTGCCACGCATGACGC-5’
4xG2T	5’-AGGTATCAGCATAATGGCCACGGTGCGTACTGCG(TTAGTG)$_4$-3’<br>3’-TCCATAGTCGTATTACCGGTGCCACGCATGACGC-5’

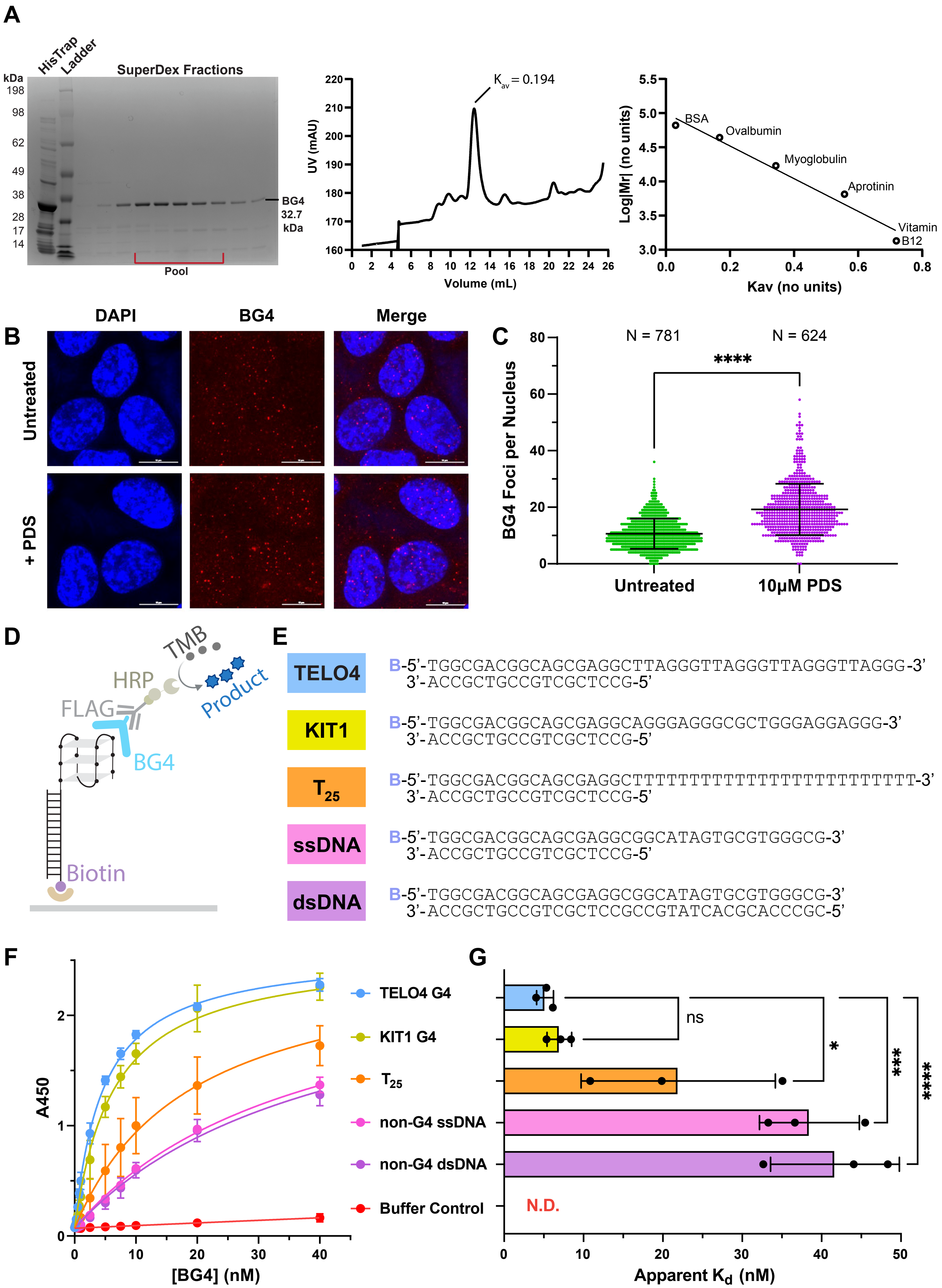
A
HisTrap
Ladder
SuperDex Fractions
kDa
198
98
62
49
38
28
17
14
BG4
32.7
kDa
Pool
K_av = 0.194
UV (mAU)
Volume (mL)
Log|Mr| (no units)
BSA
Ovalbumin
Myoglobulin
Aprotinin
Vitamin
B12
Kav (no units)
B
DAPI
BG4
Merge
Untreated
+ PDS
C
N = 781
N = 624
****
BG4 Foci per Nucleus
Untreated
10μM PDS
D
TMB
HRP
Product
FLAG
BG4
Biotin
E
TELO4
B-5'-TGGCGACGGCAGCGAGGCTTAGGGTTAGGGTTAGGGTTAGGG-3'
3'-ACCGCTGCCGTCGCTCCG-5'
KIT1
B-5'-TGGCGACGGCAGCGAGGCAGGGAGGGCGCTGGGAGGAGGG-3'
3'-ACCGCTGCCGTCGCTCCG-5'
T25
B-5'-TGGCGACGGCAGCGAGGCTTTTTTTTTTTTTTTTTTTTTTTTT-3'
3'-ACCGCTGCCGTCGCTCCG-5'
ssDNA
B-5'-TGGCGACGGCAGCGAGGCGGCATAGTGCGTGGGCG-3'
3'-ACCGCTGCCGTCGCTCCG-5'
dsDNA
B-5'-TGGCGACGGCAGCGAGGCGGCATAGTGCGTGGGCG-3'
3'-ACCGCTGCCGTCGCTCCGCCGTATCACGCACCCGC-5'
F
A450
[BG4] (nM)
TELO4 G4
KIT1 G4
T25
non-G4 ssDNA
non-G4 dsDNA
Buffer Control
G
ns
*
***
****
N.D.
Apparent Kd (nM)

Supplementary Figure 1. Validation of purified BG4 protein specificity for GQ substrates. (**A**) BG4 purification. Left panel: Coomassie-stained SDS-PAGE gel of BG4 fractions before (HisTrap Pool, lane 1) and after (SuperDex Fractions, lanes 4-12) size-exclusion chromatography. Black arrow indicates bands corresponding to BG4, with a molecular weight of about 32.7 kDa. Red bracket indicates fractions that were selected and pooled for final concentration. Middle panel: FPLC chromatogram of BG4 purification, wherein the largest peak is the BG4 elution profile. The calculated K_{av} of BG4 = 0.194. Right panel: The standard curve of the known proteins to determine the estimated molecular weight of BG4. The calculated gel phase distribution coefficient (K_{av}) is plotted against the Log_{10} values of the known molecular weights (Mr). The calculated slope is y = -2.390x + 4.997 and the estimated BG4 size = 34.3 kDa. (**B**) Representative images of BG4 foci in HeLa LT cells with and without 10 μM PDS. Fixed cells were incubated with BG4 (6 nM), which was detected with anti-FLAG immunefluorescence. Nuclei were stained with DAPI. Scale bars measure 10 μm. (**C**) Quantification of BG4 foci per nucleus. Mean and standard deviation was 10.6 ± 5.3 for untreated cells and 19.2 ± 9.1 for PDS treated cells. N= number of nuclei; **** $p<0.0001$; unpaired t-test. (**D**) Schematic of ELISA assay to detect BG4 binding to DNA constructs. (**E**) Sequences of DNA constructs with 18-base pair duplex stem. B indicates biotin. (**F**) ELISA binding curves for the indicated substrates. Binding is represented by absorbance at 450 nm vs. BG4 concentration (nM). (**G**) Apparent dissociation constants (K_d) were calculated from the nonlinear regression curves shown in (**F**). Data are mean ± SD from 3 independent experiments; ns= not significant, * $p <0.1$, *** $p<0.001$, **** $p<0.0001$; ordinary one-way ANOVA.

A

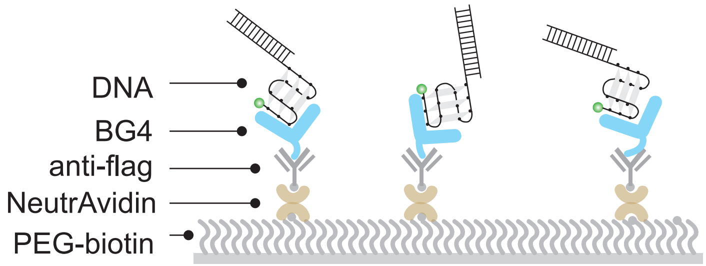

B

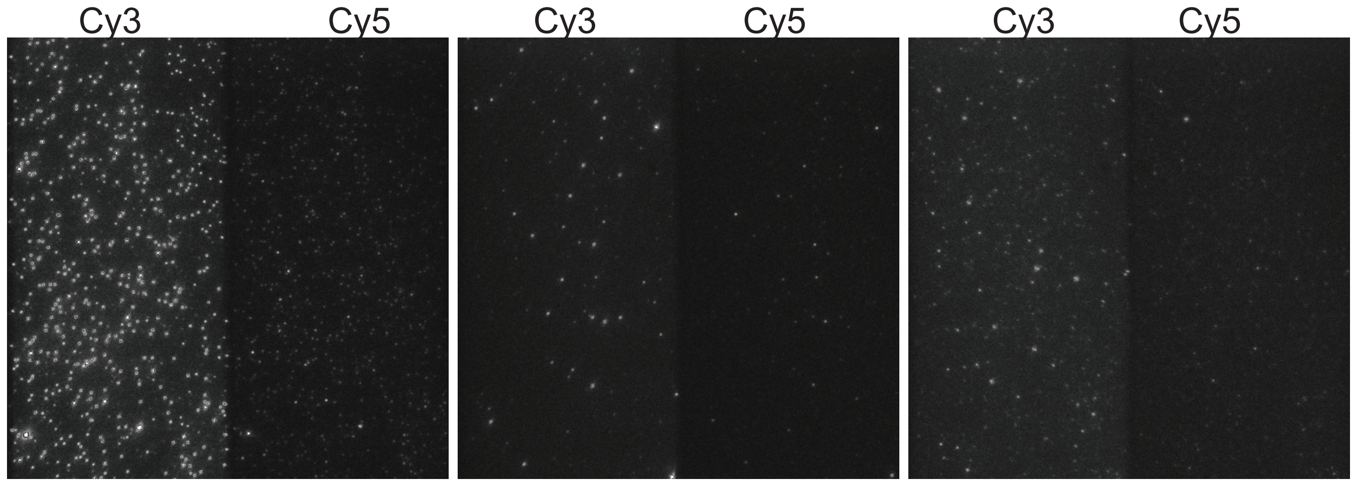

Anti-flag	+	-	+
BG4	+	+	-
TELO4	+	+	+

C

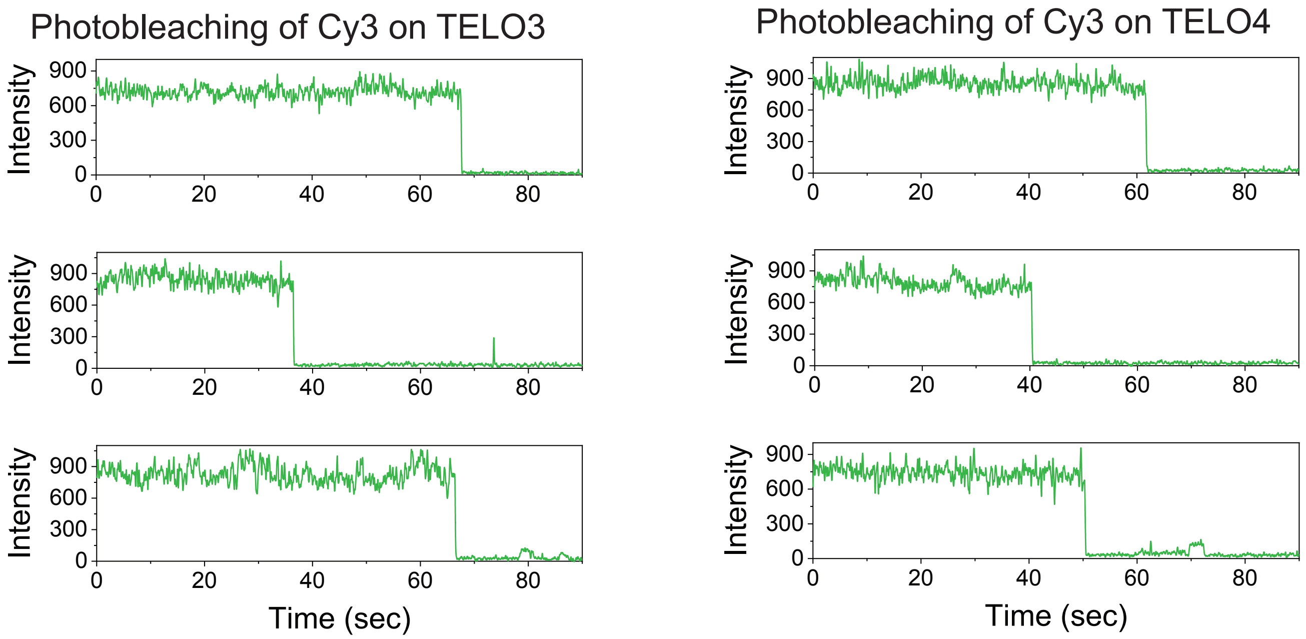

Supplementary Figure 2. Single molecule pull down assay. (**A**) Schematic of SiMPull assay in which flag-tagged BG4 was tethered to NeutrAvidin coated slide through interaction with a biotinylated anti-flag antibody. Telomeric DNA constructs were added in parallel experiments to slides prepared at the same time. (**B**) Field image view of SiMPull experiment in which the DNA is Cy3 labeled and fluorescence signal is only observed in the Cy3 channel (left) and not the Cy5 channel (right). Only background fluorescence is observed when the anti-flag antibody or BG4 protein is omitted from the experiment. (**C**) Representative single-molecule photobleacing time traces of Cy3 labeled with telomeric DNA constructs of TELO3 (left) and TELO4 (right) in SiMPull experiment. Single step photobleaching excluded bi-molecular GQ formation.

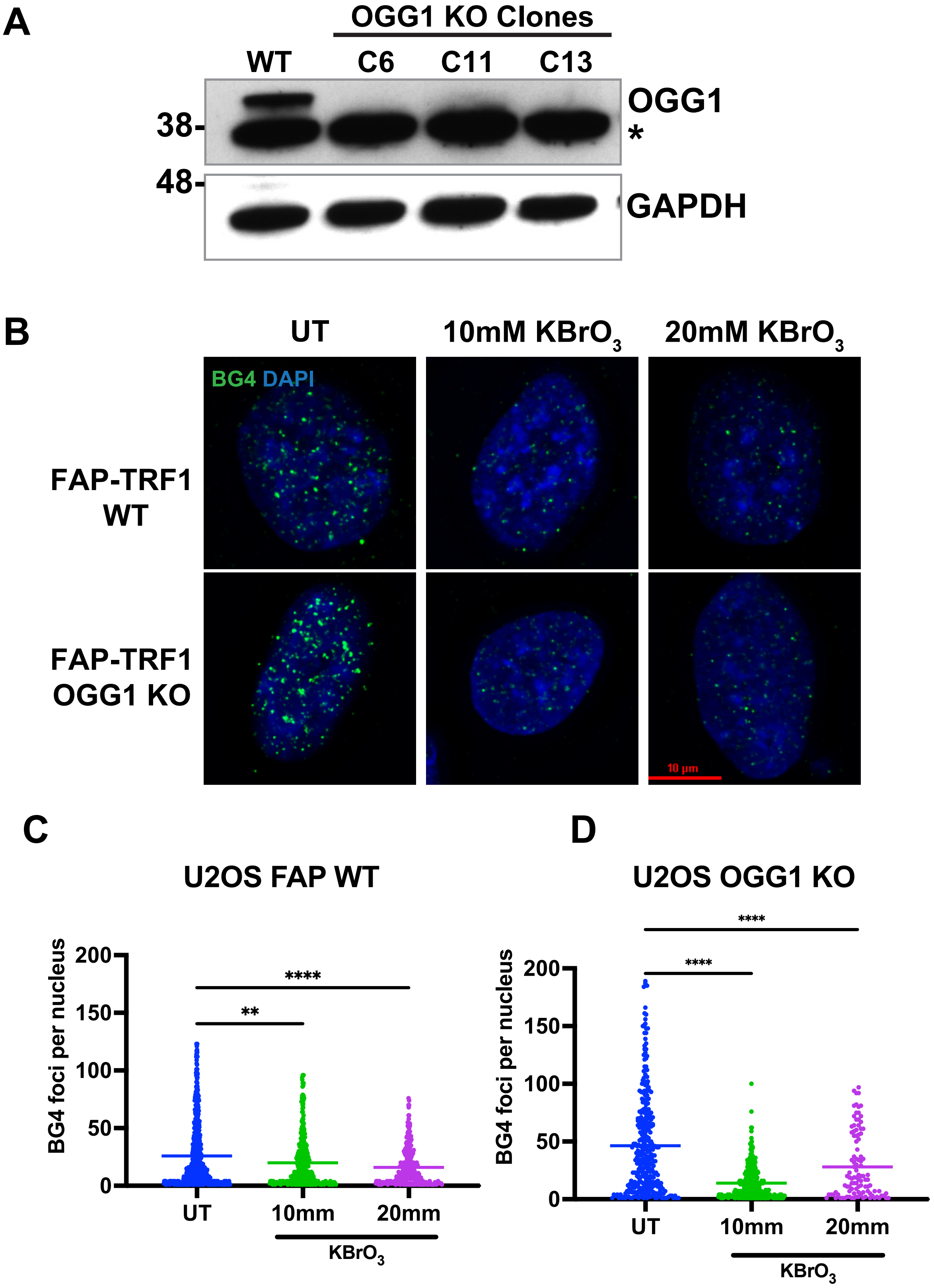

Supplementary Figure 3. Genome-wide 8-oxoG production reduces BG4 staining. (**A**) Immunoblot for OGG1 in extracts from FAP-TRF1 U2OS wild type and OGG1 knock out clones C6, C11, and C13. Arrow indicates non-specific band stained by anti-OGG1. GAPDH was used as a loading control. (**B**) Representative IF images showing BG4 (green) in FAP-TRF1 U2OS WT or OGG1 KO cells (clone 13) untreated (UT) or after 1 hour treatment with 10 mM or 20 mM KBrO3. (**C, D**). Quantification of the number of BG4 foci per nuclei analyzed; each dot represents a nucleus. Data are mean ± SD from 3 independent experiments; ordinary one-way ANOVA. $^{**}p<0.01$, $^{****}p<0.0001$.

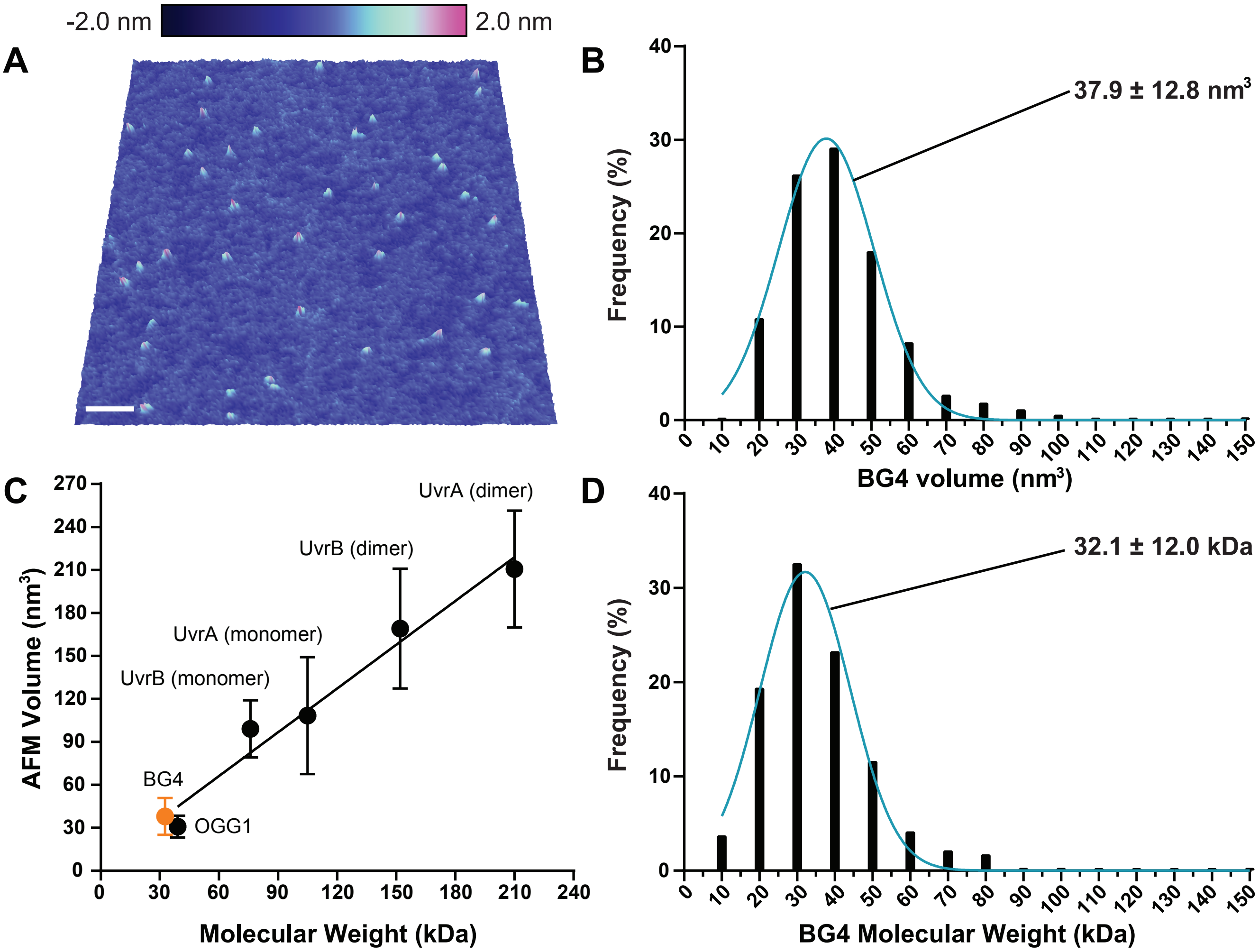

Supplementary Figure 4. BG4 is a monomer with an AFM volume that correlates with its molecular weight. (**A**) Representative three-dimensional AFM image of 8 nM BG4. White scale bar indicates 50 nm, and color scale at top represents height above surface. (**B**) Histogram of AFM volumes for BG4 particles, n = 696. (**C**) Calibration curve of AFM volume vs. molecular weight for previously characterized proteins (black) used to generate the calibration curve and BG4 (orange). Points indicate mean particle volume, and error bars show standard deviation. (**D**) Histogram of molecular weights for BG4 particles calculated from AFM. Gaussian distributions fit to the histograms are labeled with their mean ± standard deviation.
